# Avian Pathogenic *Escherichia coli* (APEC) Strain-Dependent Immunomodulation of Respiratory Granulocytes and Mononuclear Phagocytes in *CSF1R*-Reporter Transgenic Chickens

**DOI:** 10.3389/fimmu.2019.03055

**Published:** 2020-01-10

**Authors:** Andreas Alber, Katrina M. Morris, Karen J. Bryson, Kate M. Sutton, Melissa S. Monson, Cosmin Chintoan-Uta, Dominika Borowska, Susan J. Lamont, Catherine Schouler, Pete Kaiser, Mark P. Stevens, Lonneke Vervelde

**Affiliations:** ^1^Division of Infection and Immunity, The Roslin Institute and Royal (Dick) School of Veterinary Studies, University of Edinburgh, Edinburgh, United Kingdom; ^2^Department of Animal Science, Iowa State University, Ames, IA, United States; ^3^Infectiologie Santé Publique, Institut National de la Recherche Agronomique, Université de Tours, Nouzilly, France

**Keywords:** APEC, chicken, immunomodulation, macrophage, dendritic cell, heterophil, granulocyte, CSF1R

## Abstract

Avian pathogenic *Escherichia coli* (APEC) cause severe respiratory and systemic disease in chickens, commonly termed colibacillosis. Early immune responses after initial infection are highly important for the outcome of the infection. In this study, the early interactions between *GFP*-expressing APEC strains of serotypes O1:K1:H7 and O2:K1:H5 and phagocytic cells in the lung of *CSF1R*-reporter transgenic chickens were investigated. *CSF1R*-reporter transgenic chickens express fluorescent protein under the control of elements of the *CSF1R* promoter and enhancer, such that cells of the myeloid lineage can be visualized *in situ* and sorted. Chickens were separately inoculated with APEC strains expressing *GFP* and culled 6 h post-infection. Flow cytometric analysis was performed to phenotype and sort the cells that harbored bacteria in the lung, and the response of the sorted cells was defined by transcriptomic analysis. Both APEC strains were mainly detected in *CSF1R*-transgene^neg^ (*CSF1R*-tg^neg^) and *CSF1R*-tg^low^ MHC II^neg^ MRC1L-B^neg^ cells and low numbers of APEC were detected in *CSF1R*-tg^high^ MHC II^pos^ MRC1L-B^pos^ cells. Transcriptomic and flow cytometric analysis identified the APEC^pos^ *CSF1R*-tg^neg^ and *CSF1R*-tg^low^ cells as heterophils and the APEC^pos^ *CSF1R*-tg^high^ cells as macrophages and dendritic cells. Both APEC strains induced strong inflammatory responses, however in both *CSF1R*-tg^neg/low^ and *CSF1R*-tg^high^ cells, many immune related pathways were repressed to a greater extent or less activated in birds inoculated with APEC O2-*GFP* compared to APEC O1-*GFP* inoculated birds. Comparison of the immune pathways revealed the aryl hydrocarbon receptor (*AhR*) pathway, *IL17* and *STAT3* signaling, heterophil recruitment pathways and the acute phase response, are modulated particularly post-APEC O2-*GFP* inoculation. In contrast to *in vivo* data, APEC O2-*GFP* was more invasive in *CSF1R*-tg^high^ cells *in vitro* than APEC O1-*GFP* and had higher survival rates for up to 6 h post-infection. Our data indicate significant differences in the responses induced by APEC strains of prevalent serotypes, with important implications for the design and interpretation of future studies. Moreover, we show that bacterial invasion and survival in phagocyte populations *in vitro* is not predictive of events in the chicken lung.

## Introduction

APEC are the etiologic agent of colibacillosis in chickens, a complex of severe respiratory and systemic infections that constitute an important threat to all sectors of the poultry industry. Colibacillosis causes losses due to early mortality, condemnation of carcasses and reduced productivity (1). APEC constitute a large group of diverse serotypes, with the O1, O2, and O78 serogroups amongst the most prevalent globally (2).

The mechanisms underlying mucosal colonization and systemic translocation by APEC are ill-defined. Physical barriers, such as mucociliary clearance and host defense peptides may limit APEC from establishing an infection in the respiratory tract (3, 4) and the early innate immune responses, including the response by heterophils and macrophages, are thought to be important in the control of colibacillosis (5–9). In a recent study we showed that very early responses were instrumental to the fate of the birds, as chickens of a relatively susceptible inbred line succumbed to intra-air sac APEC O1:K1:H7 infection within 14 h post-inoculation whereas birds of a relatively resistant line survived (10).

Previous observational studies with strains of APEC of serogroups O1, O2, and O78 showed that APEC caused localized inflammation in the lung, often the site of onset of colibacillosis, with heterophil and macrophage recruitment to the site of inflammation within 6–12 h post-infection (5, 8), although the phenotype of the lung cells associated with APEC was never unambiguously characterized. Resistance to phagocytosis has been suggested to be an important mechanism in the development of colibacillosis. The ability of blood-derived macrophages to phagocytose and kill APEC varied between APEC strains with more virulent strains hypothesized to be able to resist the bactericidal activity of macrophages to a greater extent than less virulent strains (5, 6) or non-pathogenic avian *E. coli* (6). Several APEC virulence factors, including surface polysaccharides and fimbriae, and the K1 capsule in particular (6), were shown to contribute toward evasion of phagocytosis in birds (11). However, the role of lung phagocytes in APEC clearance and their immune response to APEC in the lung has not been previously studied.

We aimed to study the first line of cellular defense against APEC in the respiratory tract, a key porte d'entrée for pathogenic *E. coli* in poultry. We identified and phenotyped APEC-infected lung cells at 6 h post-infection (hpi) with fluorescent bacterial strains using flow cytometry and performed transcriptomic analysis on the sorted APEC-harboring cells in the lung. Owing to the large diversity of APEC strains, we inoculated chickens with sequenced strains representing two globally prevalent serotypes (O1:K1:H7 or O2:K1:H5) of the sequence-type (ST) 95 lineage, enabling us to directly compare for the first time the cellular immune response at 6 hpi in the chicken lung to these strains.

## Materials and Methods

### Chicken Line

*CSF1R*-mApple transgenic chickens (12) were provided by the National Avian Research Facility (NARF), Edinburgh, UK. All birds were hatched in a conventional animal unit and transferred to the experimental room immediately post-hatch and reared under specified pathogen-free (SPF) conditions with *ad libitum* access to feed and water. Frozen lung cells from non-transgenic Hy-Line birds (Hy-Line Brown) were used as controls for flow cytometry and cell sorting.

### Bacteria

The genome sequenced strain named APEC O1 of serotype O1:K1:H7 and ST95 was kindly provided by Professor Lisa Nolan, Iowa State University, USA [(13); GenBank NC_008563]. The strain naturally exhibits gentamicin resistance and was transformed with plasmid pFVP25.1, which constitutively expresses green fluorescent protein (*GFP*) (14) enabling APEC-infected cells to be detected by virtue of their fluorescence (APEC O1-*GFP*). The genome sequenced APEC O2 strain of serotype O2:K1:H5 and ST95 (named BEN2908; GCA_902705295 (https://www.ebi.ac.uk/ena/data/view/GCA_902705295) available at European Nucleotide Archive) was also transformed with pFVP25.1, generating strain APEC O2-*GFP* (15, 16). APEC strains were cultured overnight to stationary phase in Lysogeny Broth (LB, Luria formulation, Sigma Aldrich, UK) containing 100 μg/ml ampicillin (Sigma Aldrich, UK) to maintain pFVP25.1, with shaking at 180 rpm at 37°C. The inocula were prepared by collection of bacteria from fresh cultures by centrifugation and resuspension in sterile, apyrogenic phosphate-buffered saline (PBS) and the inoculation dose confirmed by retrospective plating of serial dilutions onto MacConkey agar (MCA) plates.

### Experimental Design

Six-week old *CSF1R*-reporter transgenic chickens were inoculated with 1 × 10^9^ colony-forming units (CFU) of APEC O1-*GFP* or APEC O2-*GFP* in 100 μl PBS or 100 μl PBS as control, administered into the right caudal thoracic air sac, and culled 6 hpi. Clinical signs and colibacillosis lesions were recorded as previously described (10) with cumulative scores based on lesions in the lungs, air sacs, liver and pericardium. Viable bacteria in the cranial right and left lung, blood, spleen, and liver were enumerated as described below. Right and left lung tissue was collected independently to prepare gradient purified lung leukocytes and an aliquot used to phenotype APEC^pos^ cells by flow cytometry in parallel to the cell sorting to obtain APEC^pos^ cells for transcriptomic analysis. Two birds per day were inoculated due to the time and resources required for downstream processing. A total of 22 birds from three hatches were used for the APEC O1-*GFP* studies (APEC O1-*GFP, n* = 13; PBS controls, *n* = 9). A total of 24 birds from two hatches were used for the APEC O2-*GFP* studies (APEC O2-*GFP, n* = 12; PBS controls, *n* = 12).

### Bacteriological Analysis of Tissues

Viable bacteria in tissues were enumerated as previously described (10), with minor changes. APEC O1-*GFP* samples were plated onto MCA plates containing 10 μg/ml gentamicin (ThermoFisher, UK). APEC O2-*GFP* samples were plated onto antibiotic-free MCA plates. To account for potential unrelated bacterial contaminants and for potential loss of pFVP25.1 within 6 hpi in birds, all right lung samples from birds inoculated with both APEC strains were additionally plated onto antibiotic-free LB agar plates and their fluorescence confirmed under UV light. All obtained colonies were *GFP* positive (not shown).

### Preparation of Lung Leukocytes for RNAseq

After collecting cranial lung tissue to enumerate viable bacteria, the right and left lungs were collected separately in 5 ml PBS with 100 μl heparin (5,000 units/ml, Wockhardt, UK) and 50 μl RNAse inhibitor (0.4 units/μl RNAsin Plus, Promega, UK). Maintaining the tissue in collection buffer with RNAse inhibitor (0.4 units/μl), tissues were cut into small pieces and enzymatically digested with an equal mix of DNAse I and collagenase A (1 and 3 mg/ml, respectively, Sigma Aldrich, UK) in supplement-free RPMI 1640 media (Sigma Aldrich, UK) for 30 min at 37°C and 5% CO_2_. The digested lung tissue was passed through a 70 μm cell strainer on ice. Samples were washed once with cold PBS (350 x g, 5 min, 4°C) and lung leukocytes obtained by gradient purification with Histopaque 1.077 (Sigma Aldrich, UK) for 20 min at 400 x g at room temperature (RT). The interface and layer above were collected and the samples washed twice with cold PBS. The cell numbers and viability were determined by Trypan Blue staining (Corning, UK) and the cells re-suspended in cell sorting buffer (PBS with 0.5% bovine serum albumin, BSA; Sigma Aldrich, UK) with RNAse inhibitor.

### Flow Cytometry and Cell Sorting

Flow cytometry was performed with a BD LSRFortessa™ (BD Biosciences, UK) flow cytometer in parallel to the cell sorts as previously described (10). All gate settings were based on fluorescence minus one (FMO) and isotype-matched controls using previously isolated and frozen lung cells from non-transgenic Hy-Line and *CSF1R*-reporter transgenic chickens. The following gating strategy was applied: single cells, live cells, *CSF1R*-tg^neg^, *CSF1R*-tg^low^, or *CSF1R*-tg^high^ cells, and results are expressed as percentage thereof by using FlowJo^®^ 10.4 (FlowJo, US). The following antibodies were used: mouse anti-chicken CD45 (clone UM16-6), mouse anti-chicken CD3 (clone CT-3), mouse anti-chicken chB6 (Bu-1; clone AV20), and mouse anti-chicken MRC1L-B (KUL01) all purchased from Bio-Rad, UK; mouse anti-chicken MHC II (clone 2G11, Abcam, UK); mouse anti-chicken putative CD11 (clone 8F2) and mouse anti-chicken K1 (both kind gifts from Dr. S. Härtle, LMU, Germany); mouse anti-chicken GRL1 and mouse anti-chicken GRL2 (Developmental Studies Hybridoma Bank, University of Iowa, US); and goat anti-mouse IgG1:Alexa Fluor^®^647 (AF647), goat anti-mouse IgG2a:AF647, and goat anti-mouse IgG3:AF647 all from ThermoFisher, UK. All samples were stained with SYTOX^TM^ Blue (ThermoFisher, UK) for live cell gating.

APEC^pos^ lung cells were sorted with a BD FACS ARIA IIIu (BD Biosciences, UK) cell sorter. Three cell populations were sorted from the APEC-inoculated birds, with all gate settings based on single, live cells gated similar as for the flow cytometry performed in parallel. The *CSF1R*-tg^low^ and *CSF1R*-tg^neg^ cell populations that harbored APEC could not be distinguished from each other with sufficient accuracy and were therefore sorted as one population (*CSF1R*-tg^neg/low^). From the APEC-inoculated birds the APEC^pos^ *CSF1R*-tg^high^, APEC^neg^ *CSF1R*-tg^high^, and APEC^pos^ *CSF1R*-tg^neg/low^ cells were sorted. From the PBS control birds, the *CSF1R*-tg^high^ cells were collected. All cell populations were collected in cold cell sorting buffer with RNAse inhibitor and processed immediately post-sort. For RNA isolation, the samples were centrifuged for 10 min at 400 x g and 4°C, cell pellets lysed in RLT lysis buffer with β-mercaptoethanol (QIAGEN, UK) and stored at −80°C.

### RNA Sequencing

Pilot RNA extractions showed that a minimum of 4 x 10^4^ cells were required from the APEC^pos^ *CSF1R*-tg^high^ cell populations and 1.5 × 10^5^ cells from the APEC^pos^ *CSF1R*-tg^neg/low^ cell populations to obtain sufficient total RNA for cDNA generation. To achieve these cell numbers the left and right lung samples of the same bird were pooled (Supplementary Table 1). Additionally, combining samples from two birds where required did not alter the final analysis as described below. A minimum of 4 and maximum of 6 samples per sorted cell population were submitted for RNAseq (Supplementary Tables 1, 2).

RNA from sorted cells was extracted using the RNeasy micro kit (QIAGEN, UK) and concentrated using the SpeedVac RC1022 (ThermoFisher, UK). RNA quantity and quality (RNA integrity index > 7.5) was assessed by high sensitivity Agilent RNA ScreenTape assay with the TapeStation 2200. cDNA was amplified with the Ovation RNAseq System v2 kit (NuGen, UK) using 7 ng total RNA with all APEC O1-*GFP* and 3.5 ng total RNA with all APEC O2-*GFP* samples. TruSeq DNA Nano gel free libraries (350 bp insert, Illumina, UK) were prepared and sequenced on the NovaSeq S4 (Illumina, UK), yielding at least 68 M total 75 bp paired-end reads per sample. All procedures were performed according to manufacturer's instructions.

### Transcriptomic Analysis

Obtained reads were trimmed using Trimmomatic [version 0.36, (17)] to remove adaptor sequences of the TruSeq DNA Nano kit and for quality. Reads after trimming were required to have a minimum length of 50 bases. The RNAseq reads were mapped to the reference genomes using STAR aligner software package [version 2.5.1b, (18)]. Reads were initially annotated to the respective APEC genome, and subsequently to the *Gallus gallus* (Gallus_gallus-5.0) Ensembl reference genome (annotation version 84). Raw counts for each annotated gene were obtained using the feature counts software [version 1.5.2, (19)].

Differential gene expression analysis was performed within the Bioconductor edgeR package [version 3.16.5, (20)]. Statistical assessment of differential expression was carried out with the likelihood-ratio test. Contrasts specified were pairs of sorted cell populations within APEC O1-*GFP* or APEC O2-*GFP* (APEC^pos^ *CSF1R*-tg^high^, APEC^pos^ *CSF1R*-tg^neg/low^, APEC^neg^ *CSF1R*-tg^high^, PBS *CSF1R*-tg^high^). Differentially expressed genes were defined as those with a false-discovery rate (FDR) < 0.05 and log_2_ fold-change (FC) > 2. Heatmaps were constructed in R using the pheatmap package (v. 1.0.10; https://CRAN.R-project.org/package=pheatmap). Over-representation of gene ontology (GO) terms was investigated using the PANTHER Over-representation Test [released 20171205; (21)] using Fisher's Exact with FDR multiple test correction. Chicken gene symbols were converted to the orthologous human symbols and analyzed using the human PANTHER database. Network analysis for both sample-sample network and gene-gene network was performed in BioLayout 3D (22) which performs a Pearson correlation matrix calculated for each pair of samples or genes, using a modified Fruchterman-Rheingold algorithm. Clustering was performed on these networks using the Markov clustering algorithm (MCL) with an inflation value of 1.8. For graphical purposes, clusters in the gene-gene network graph were required to have a minimum of 30 nodes, and clusters showing even expression across treatment groups or associated with gender were removed. The Ingenuity Pathway Analysis (IPA) program (QIAGEN, UK) was used to identify cellular canonical pathways and physiological functions (*P* ≤ 0.05 and *Q* ≤ 0.05).

Combining samples from two birds where required did not alter the final analysis, as evidenced by processing of individual and pooled samples from four APEC O1-*GFP* inoculated samples with sufficient RNA yield (Bird 8, 9, 10, and 19). Transcriptomic analysis of these samples was performed identically to the other study samples and as described above with the result of zero significantly differentially expressed genes (DEGs) between groups with either individual or pooled samples.

### Phagocytosis and Killing Assays

Phagocytosis and killing assays were performed as previously described (10). In short, lung cells from 6- to 8-week-old *CSF1R*-reporter transgenic birds were isolated using gradient purification and the freshly isolated cells in suspension were inoculated with APEC O1-*GFP* or APEC O2-*GFP* in stationary phase at a multiplicity of infection (MOI) of 10 for 30 min at 41°C, after which extracellular bacteria were killed by addition of 500 μg/ml ceftazidime hydrate (Sigma Aldrich, UK) for 30 min. Then, the cells were collected (0 h, phagocytosis and invasion) or incubated for a further 2, 4, or 6 h to enumerate viable bacteria. The same sample was analyzed by flow cytometry to phenotype the APEC^pos^ cells. To determine whether the bacteria actively invaded or were phagocytosed the same experimental settings were used, using live and heat-killed bacteria and cells were collected at 0 hpi. The bacteria were killed by incubation at 56°C for 24 h in a water bath. *GFP* expression was checked for each experiment prior to inoculation and was not affected by heat treatment. Two independent phagocytosis and killing assays were performed (7 birds in total). Phagocytosis of live and killed bacteria was compared in two studies (4 birds in total).

### Confocal Microscopy

Primary lung *CSF1R*-tg^high^ mononuclear phagocytes were obtained from 6- to 8-week-old *CSF1R*-reporter transgenic birds by virtue of their adherence to plastic during culture as previously described (23). Cells were seeded at 7 x 10^6^ cells on Nunc Lab-Tek 4-well chamber slides (ThermoFisher, UK). After 24 h culture, cells were washed twice with PBS followed by inoculation with APEC O1-*GFP* or APEC O2-*GFP* with an MOI of 10 for 30 min at 41°C, 5% CO_2_. Extracellular bacteria were killed by 500 μg/ml ceftazidime hydrate treatment for 30 min. Cells were washed twice with cold PBS and fixed with 4% paraformaldehyde for 20 min on ice. Cells were stained with mouse anti-chicken MRC1L-B (clone KUL01, Bio-Rad, UK) and mouse anti-chicken MHC II (clone 2G11, Southern Biotech, UK) followed by goat anti-mouse IgG1:AF647 and counterstained with 4',6-diamidino-2-phenylindole (DAPI). All antibodies were diluted in PBS supplemented with 1% BSA and 0.5% Triton X-100 (Sigma Aldrich, UK) and incubated on ice for 1 h. For cell images and 3D rendering, Z-stacks were obtained using an inverted laser-scanning microscope (LSM) 710 (Zeiss, UK), 40X or 63X Nikon oil lenses and images were captured using ZEN 2012 software (black edition, Carl Zeiss, UK) and analyzed using ZEN 2012 (Blue edition, Zeiss, UK) or Imaris software (version 9.3, Bitplane, Switzerland).

### Statistical Analysis

All data were not normally distributed and were therefore analyzed by Mann Whitney tests for the APEC comparison throughout the study, using GraphPad Prism 7.00 (GraphPad, US). The probability level for significance was taken as *P* ≤ 0.05. Statistical tests performed for the transcriptomic analysis are outlined in the relevant methods section.

## Results

### APEC O1-*GFP* and APEC O2-*GFP* Cause Systemic Infection Within 6 h Post-inoculation

**Figure 1:**
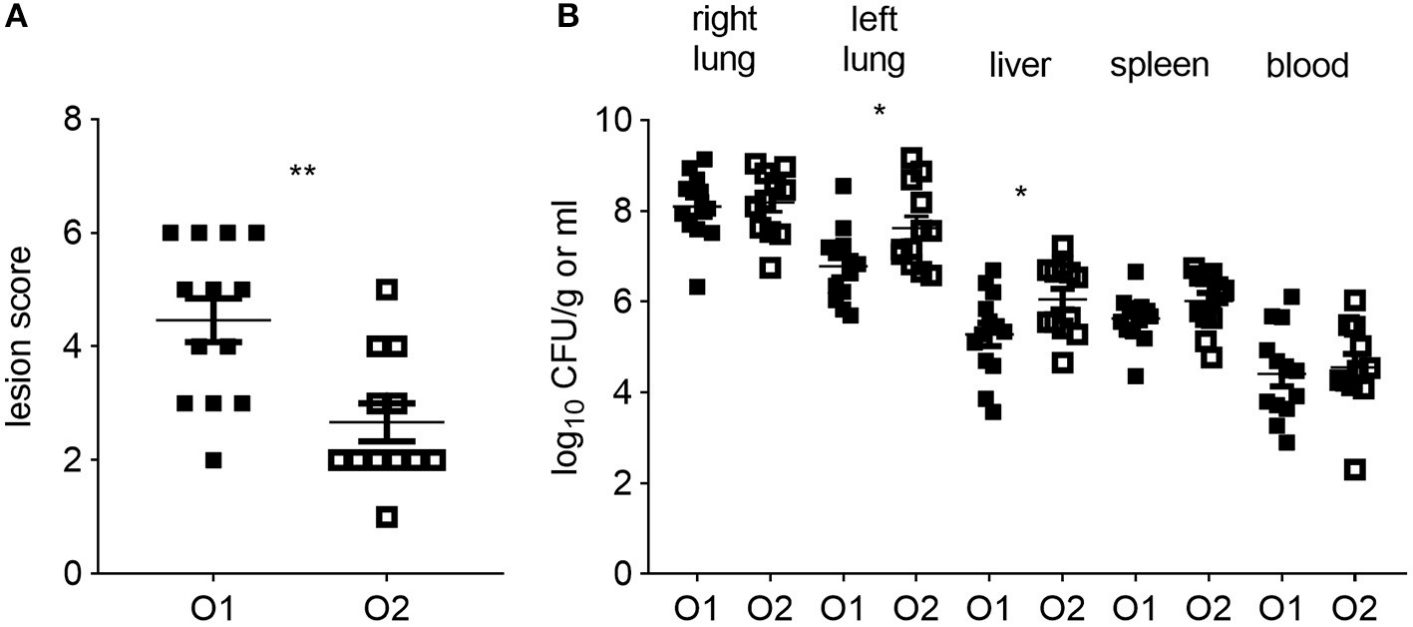
Colibacillosis lesion scores and bacterial colonization of tissues post-APEC inoculation. **(A)** Colibacillosis lesion scores of APEC-inoculated birds and **(B)** bacterial loads in right and left lung, liver, spleen and blood of APEC-inoculated birds. O1 is APEC O1-*GFP*, O2 is APEC O2-*GFP*, tissues as indicated on graphs. *n* = 13 APEC O1-*GFP, n* = 12 APEC O2-*GFP*. The mean with standard error of the mean (SEM) is shown. ^*^P < 0.05, ^**^P < 0.01.

All *CSF1R*-reporter transgenic birds inoculated with either strain of APEC were examined for clinical signs and macroscopic lesions. Clinical signs were limited to hunched posture at 6 hpi and no differences were seen between the APEC O1-*GFP* and APEC O2-*GFP* inoculated birds (not shown). In contrast, the macroscopic lesion scores were significantly lower in the APEC O2-*GFP* inoculated birds compared to the birds inoculated with APEC O1-*GFP* (Figure 1A; *P* = 0.0028). Inoculation with APEC O1-*GFP* or APEC O2-*GFP* resulted in rapid systemic dissemination and bacterial loads typically in excess of 10^4^ CFU per ml or gram were detected in blood, spleen and liver. The bacterial loads in the right lung, spleen and blood did not significantly differ between the strains. However, in the left lung, opposite the inoculation site, and liver, the bacterial loads were significantly higher after APEC O2-*GFP* inoculation compared to inoculation with APEC O1-*GFP* (Figure 1B; *P* = 0.0398 and 0.0411, respectively).

### Phenotypic Characterization of the Host Cells Harboring APEC

**Figure 2:**
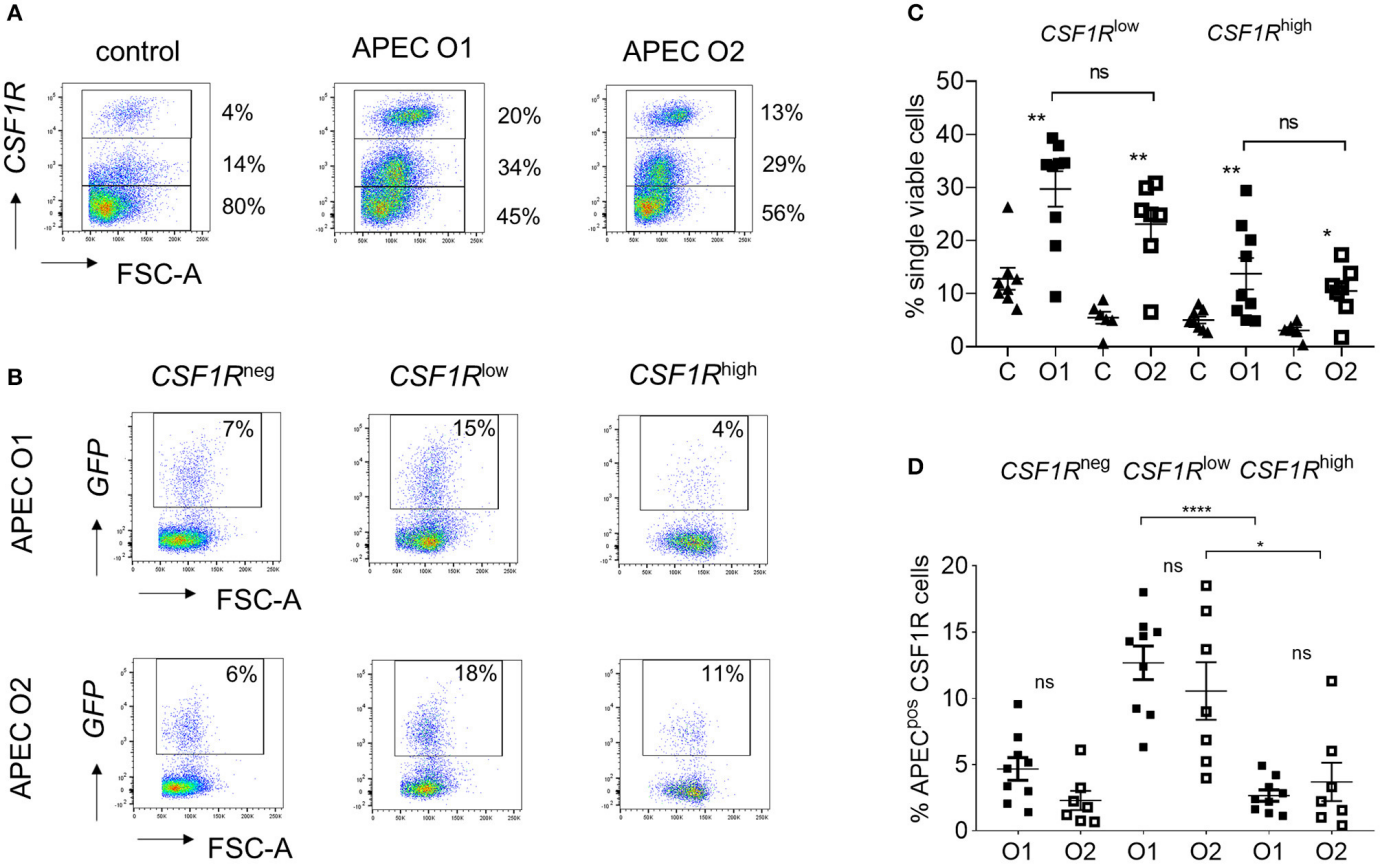
Flow cytometric analysis of single, live lung cells from APEC inoculated birds. Gradient purified lung cells were isolated and subjected to flow cytometric analysis to detect APEC^pos^ cells. **(A)** Representative plots showing the increase of *CSF1R*-tg^high^ and *CSF1R*-tg^low^ cell numbers within 6 hpi, **(B)** quantification of the increase of *CSF1R*-tg^high^ and *CSF1R*-tg^low^ cell numbers amongst all birds, **(C)** representative plots showing the APEC^pos^ cells in *CSF1*-tg^neg^, *CSF1R*-tg^low^, and *CSF1R*-tg^high^ cells of a representative APEC O1-*GFP* and APEC O2-*GFP* inoculated bird and **(D)** quantification of the APEC^pos^ cells in *CSF1R*-tg^neg^, *CSF1R*-tg^low^, and *CSF1R*-tg^high^ cells amongst all birds. O1 is APEC O1-*GFP*, O2 is APEC O2-*GFP*. *n* = 9 APEC O1-*GFP, n* = 7 APEC O2-*GFP*. The mean with SEM is shown. ^*^P < 0.05, ^**^P < 0.01, ^****^P < 0.0001.

To determine the phenotype of the cells harboring APEC O1-*GFP* or APEC O2-*GFP*, the lung cells were analyzed by flow cytometry 6 hpi. Flow cytometric phenotyping of APEC O1-*GFP* or APEC O2-*GFP* positive cells revealed similar results. Both strains were detected in CD45^pos^ leukocytes (Supplementary Figure 1). The *CSF1R*-transgene population could be divided into *CSF1R*-tg^low^ and *CSF1R*-tg^high^ populations (Figure 2A). The *CSF1R*-tg^low^ and *CSF1R*-tg^high^ cell populations significantly increased relative to control birds at 6 hpi, with no significant difference observed between the APEC strains (Figures 2A,B). Phenotypic characterization indicated that the bacteria were primarily found in *CSF1R*-tg^low^ and *CSF1R*-tg^neg^ cells, with no significant difference between the APEC strains (Figures 2C,D). These APEC^pos^ *CSF1R*-tg^low^ and *CSF1R*-tg^neg^ cells expressed CD45, CD11, GRL1, and GRL2 but not T or B cell markers, CD3 and chB6, and lacked MHC II expression or markers expressed on monocytes, macrophages and thrombocytes, such as MRC1L-B, and K1. In contrast, APEC^pos^ *CSF1R-*tg^high^ cells expressed CD45, CD11, GRL1, GRL2, MRC1L-B, and MHC II, were K1^−/low^ and lacked surface expression of CD3 and chB6 (Supplementary Figures 1A–F).

**Figure 3:**
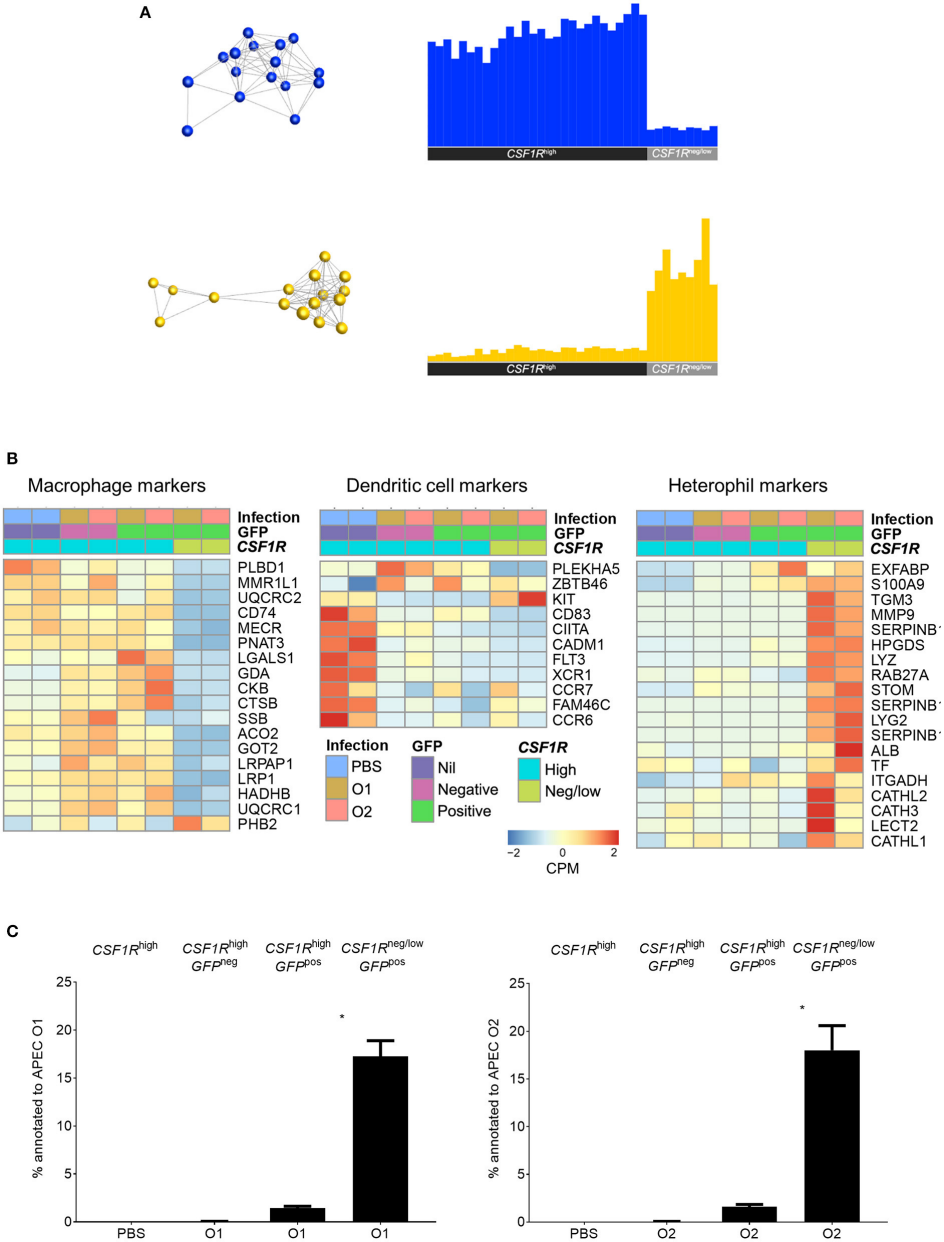
Transcriptome analysis of sorted lung cells from uninfected and APEC infected birds. **(A)** Network graph of macrophage (blue) and heterophil (yellow) marker expression with the corresponding graph of the average expression of the markers across all samples. This macrophage and heterophil related genes each formed single clusters that showed high expression in the *CSF1R*-tg^high^ and *CSF1R*-tg^neg/low^ samples, respectively. **(B)** Heatmaps of normalized expression of macrophage, DC and heterophil related genes cross the sorted cells post-infection. Heatmap coloring is based on counts per million with red indicating high counts and blue indicating low counts. **(C)** Annotation of the obtained reads of APEC O1 or APEC O2 genome from the RNA-seq data from the sorted cells showing a significantly higher percentage of bacterial reads in the APEC^pos^ *CSF1R*-tg^neg/low^ cells as compared to the APEC^pos^ *CSF1R*-tg^high^ cells, and no difference between the APEC strains. The mean with SEM is shown. ^*^P < 0.05.

For transcriptomic analysis the APEC^pos^ and APEC^neg^ cells were collected based on their *CSF1R*-transgene expression. The *CSF1R*-tg^low^ and *CSF1R*-tg^neg^ cell populations that harbored APEC were sorted as one population as they could not be distinguished from each other with sufficient accuracy and named *CSF1R*-tg^neg/low^. The other populations were sorted based on their APEC^pos^ *CSF1R-*tg^high^ and APEC^neg^ *CSF1R-*tg^high^ phenotype, in addition to *CSF1R-*tg^high^ cells from PBS-inoculated control birds. To establish the nature of the APEC^pos^ cells, transcriptomic analysis was performed. Using previously published data on key macrophage and heterophil gene markers in the chicken [20 markers for each population (24)] we compared the expression of these genes between the APEC^pos^ *CSF1R-*tg^neg/low^ and APEC^pos^ *CSF1R*-tg^high^ cells for both APEC O1-*GFP* and APEC O2-*GFP* inoculated birds. The APEC^pos^ *CSF1R*-tg^high^ cells had high expression of macrophage related genes and low expression of heterophil related genes and *vice versa* for the APEC^pos^ *CSF1R-*tg^neg/low^ cells (Figure 3A). Heatmaps were generated and included DC related genes which further revealed that the *CSF1R*-tg^high^ cells from PBS-inoculated control birds contained both macrophages and dendritic cells, whilst the *CSF1R*-tg^high^ cells post-APEC O1-*GFP* or APEC O2-*GFP* inoculation were enriched for macrophage related genes, with higher expression levels of macrophage genes (*LGALS1, GDA, CKB, CTSB, LRPAP1, LRP1, HADHB, UQCRC1*) and lower expression levels of most DC related genes (*KIT, CD83, CIITA, CADM1, FLT3, XCR1, CCR7, FAM46C, CCR6*) (Figure 3B).

Although flow cytometric analysis detected the cells harboring APEC, the bacterial load could not be determined in the cell subpopulations by direct plating. The transcriptome data revealed that APEC^pos^ *CSF1R-*tg^neg/low^ cells had a 10-fold higher percentage of the reads annotated to the APEC O1:K1:H7 or APEC O2:K1:H5 genome, as compared to the APEC^pos^ *CSF1R*-tg^high^ cells (Figure 3C).

### Overview of Relationships in the Transcriptome Data

**Figure 4:**
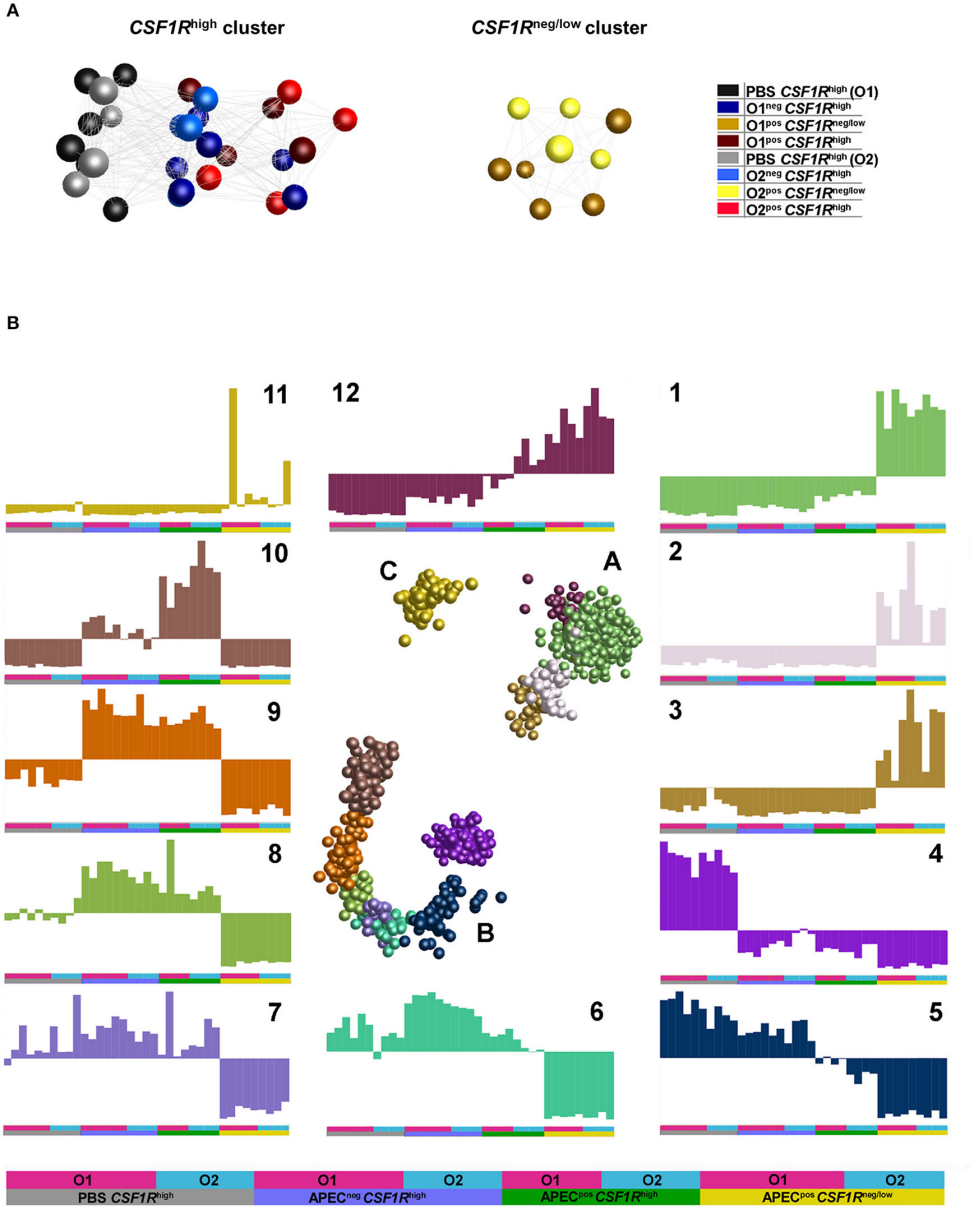
Network graphs of gene expression (normalized counts per million). **(A)** Sample-sample network graph. The graph is colored based on experimental groups and shows primary separation of samples based on *CSF1R*-transgene expression. **(B)** Gene-gene network graphs. The graph is colored by clusters determined by MCL clustering, with three separated clusters of genes (A–C) comprised of 12 separate clusters. The expression of each cluster is shown in the graphs labeled 1–12, with the experimental groups denoted by a key at the base of the figure and used under each graph.

An unbiased sample-sample network was produced using BioLayout 3D. This indicated that the main division of samples is between the *CSF1R-*tg^neg/low^ and *CSF1R-*tg^high^ cells (Figure 4A). In the *CSF1R*-tg^high^ cluster, a division between PBS-inoculated and APEC-inoculated birds was observed, and the APEC^neg^ *CSF1R*-tg^high^ samples from infected birds were positioned between the PBS-inoculated and APEC^pos^ *CSF1R*-tg^high^ samples. This suggests that the APEC^neg^*CSF1R-*tg^high^ cells in infected birds had a bystander response and were activated without harboring bacteria (Figure 3B). The APEC O1-*GFP* and APEC O2-*GFP* infected cells for each cell type do not show a clear separation in the graph for either *CSF1R*-tg^high^ or *CSF1R-*tg^neg/low^ groups (Figure 4A).

To examine the main trends in the data, an unbiased gene-gene network graph with Markov clustering was constructed (Figure 4B). Three main groups of genes were observed, the largest group (B) showed elevation in the *CSF1R*-tg^high^ samples, the second largest (A) showed elevated expression in the *CSF1R-*tg^neg/low^, and the smallest group (C) showed elevated expression in only a few of *CSF1R-*tg^neg/low^. One cluster showed elevated expression in the *CSF1R-*tg^neg/low^ samples (cluster 1, 930 genes), and this was enriched for immune signaling pathways (determined by GO overrepresentation test) including TLR, interleukin, apoptosis and PDGF signaling pathways (Supplementary Table 3). Key genes in cluster 1 included AMPs (*AvBD3, DEF6*), cytokines and cytokine receptors (including *CCL4, CSF3R, CXCL12, CXCR1, FASLG, FAS, IFNAR1, IFNGR2, IL16, IL17C, IL1B, IL8, TNFRSF10B, TNFRSF21, TNFSF11*), TLRs (*TLR15, TLR4*), JAK/STAT genes (*JAK1, JAK3*), ISGs (including *IFITM10, IRF7, IRF2, IFI6, RSAD2*) and immune cell receptors and markers (including *CD247, CD55, CHIR-AB1, CTSD, LYZ*). The cluster with elevated expression in the *CSF1R*-tg^high^ samples from uninfected birds (cluster 4, 251 genes) was a heterogeneous group of genes enriched only for genes associated with biological regulation (Supplementary Table 3). The cluster contained 17 DC-related genes including *FLT3, BCL11A, CADM1, CIITA, CX3CR1, VCAM1*, and *XCR1*. The cluster with elevated expression in the *CSF1R-*tg^high^ samples from infected birds independent of whether the cells were APEC^pos^ (cluster 9, 99 genes), was enriched for genes associated with MHC I antigen processing (including *CALR, CYBB, AP2M1*) and many proteasome genes, and protein metabolism/catabolism process genes. Finally, the cluster with elevated expression specifically in the APEC^pos^ *CSF1R*-tg^high^ samples (cluster 10, 161 genes) was enriched for pathways associated with regulation of response to stimulus, regulation of cell migration and adhesion, and metabolism. It also included a high proportion of cytokines, particularly those associated with immune regulation and anti-inflammatory effects (including *CXCL13, CXCL13L2, CXCL8, IL10, IL13RA2, IL19, IL20RA*, and *IL4I1*).

### Transcriptomic Response of APEC Harboring *CSF1R-*tg^high^ Cells

**Figure 5:**
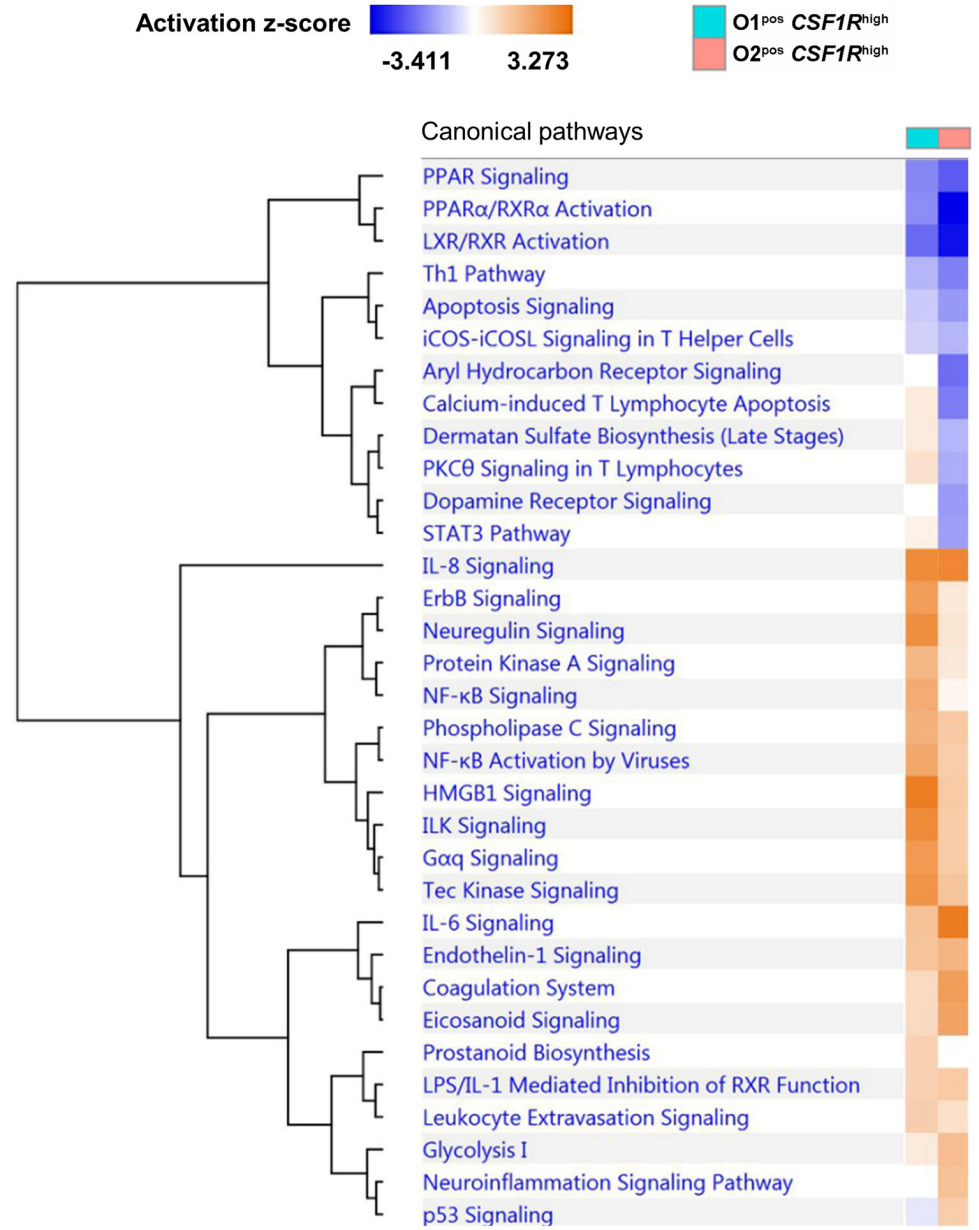
Comparative pathway analysis of APEC O1-*GFP* vs. APEC O2-*GFP* infected *CSF1R*-tg^high^ cells based on the log fold-changes of genes differentially expressed compared to *CSF1R*-tg^high^ cells from PBS control samples. Coloring shows enrichment of pathways in APEC-bearing samples compared to PBS controls (blue = pathway suppressed, orange = pathway activated). A total of 1374 (O1) and 1405 (O2) genes were included in the analysis.

We examined pathway enrichment between the *CSF1R*-tg^high^ APEC O1-*GFP* and O2-*GFP* infected cells. In general, the response of APEC O1-*GFP*^pos^ *CSF1R*-tg^high^ and APEC O2-*GFP*^pos^ *CSF1R*-tg^high^ cells to infection was very similar, with mostly the same pathways activated or repressed compared to cells from uninfected controls (Figure 5). This included involvement of many inflammatory pathways including IL-8 and IL-6 signaling and the T_h_1 pathway, suggesting a robust immune response to infection by both APEC strains. However, there were numerous instances of pathways that were repressed to a greater extent during APEC O2-*GFP* infection relative to uninfected birds, when compared to APEC O1-*GFP* infection. For example, the “calcium-induced T Lymphocyte Apoptosis,” “Aryl Hydrocarbon Receptor (AhR) Signaling,” “STAT3 Pathway,” and “PCK theta signaling in T lymphocytes” pathways were repressed to a greater extent during APEC O2-*GFP* infection (Figure 5). Moreover, we found instances of pathways that were less activated in APEC O2-*GFP*^pos^ cells relative to controls than observed during APEC O1-*GFP* infection, including “Neuregulin signaling and ErbB,” “NF-κB signaling,” and “HMGB1 and ILK signaling.” Neuregulins (NRGs) are a family of structurally related signaling proteins that bind to receptor tyrosine kinases of the ErbB family and mediate a myriad of cellular functions including survival, proliferation, and differentiation in both neuronal and non-neural cells including macrophages. *HMGB1* is secreted by immune cells and activated macrophages and monocytes secrete *HMGB1* as a cytokine mediator of inflammation.

**Figure 6:**
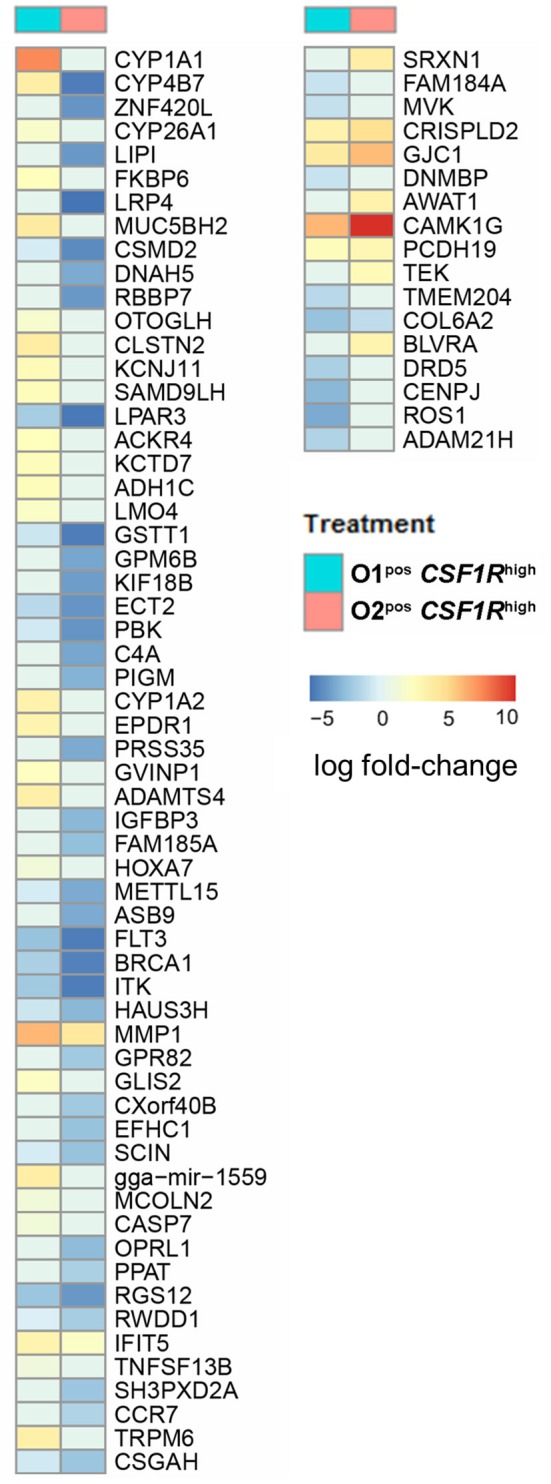
All annotated DEGs from the contrast of APEC O1-*GFP* vs. APEC O2-*GFP* infected *CSF1R*-tg^high^ cells. Coloring is based on fold-change expression of APEC-bearing cells compared to their PBS controls, with red indicating higher expression and blue lower expression.

Strain-specific responses were analyzed by comparison of the genes that were differentially expressed in APEC O1-*GFP*^pos^ *CSF1R*-tg^high^ and APEC O2-*GFP*^pos^ *CSF1R*-tg^high^ cells. A hundred DEGs, of which 77 are annotated with gene symbols, were observed (Supplementary Table 4). This group of 77 genes had an over-representation of genes related to cell signaling and communication. The differences between the APEC O1-*GFP* and APEC O2-*GFP* infected *CSF1R*-tg^high^ cells were generally due to downregulation of gene expression in APEC O2-*GFP* samples, as seen when examining log fold-changes of infected samples to PBS controls (Figure 6). The genes showing the greatest difference between the two APEC strains were *CYP1A1*, which is a key component of AhR signaling, a chicken homolog of *ZNF420* which regulates apoptosis, *FKBP6* which has a role in immunoregulation, and two additional cytochrome P450 family members (*CYP4B7, CYP26A1*). Additional key genes that showed downregulation in APEC O2-*GFP* infected samples were *CYP1A2*, also part of the AhR signaling pathway, the complement component *C4A* and a protein involved in complement control (*CSMD2*), a gene key in apoptosis response (*CASP7*), and a homolog of *SAMD9L*, a gene involved in the regulating proliferation and maturation of blood cells. There were also two DC related genes that showed significant difference between O1 and O2 (*FLT3* and *CCR7*); these were downregulated following both APEC O1-*GFP* and APEC O2-*GFP* infection, but to a greater extent in the APEC O2-*GFP* infected cells.

### Transcriptomic Response of APEC Harboring *CSF1R-*tg^neg/low^ Cells

Lung *CSF1R*-tg^low^ cells from uninfected birds were sorted and analyzed in an independent study. As expected, these cells formed a heterogenous group of cells, including heterophils, B cells and T cells (data not shown). Since there is no relevant uninfected control for the *CSF1R*-tg^neg/low^ samples the only comparison that could be made was directly between APEC O1-*GFP*^pos^ and APEC O2-*GFP*^pos^ *CSF1R*-tg^neg/low^ cells. Between these cell populations, 91 genes showed differential expression, 59 of which were annotated, suggesting that the overall response of APEC O1-*GFP* and APEC O2-*GFP* infected *CSF1R*-tg^neg/low^ cells are similar. In line with the transcriptomic analysis of *CSF1R*-tg^high^ cells, both APEC strains induced an inflammatory response, but many genes showed lower expression after APEC O2-*GFP* inoculation, in a direct comparison between APEC O1-*GFP*^pos^ and APEC O2-*GFP*^pos^ *CSF1R*-tg^neg/low^ cells (Supplementary Table 4). Two genes with the highest gene count difference between the APEC strains were involved in apoptosis, *DTHD1* and *EVA1A*, suggesting that apoptosis may be modulated differently between the APEC strains. The other genes showing the largest difference with higher expression in APEC O1-*GFP* infected samples, were *FABP2* which is involved in fatty acid metabolism, a chicken-specific chemokine receptor (*CCR8L*), two cytokines (*CCL19, IL22*), *TRPC6*, which is involved in calcium channel activity, and the *SAMD9L* homolog.

**Figure 7:**
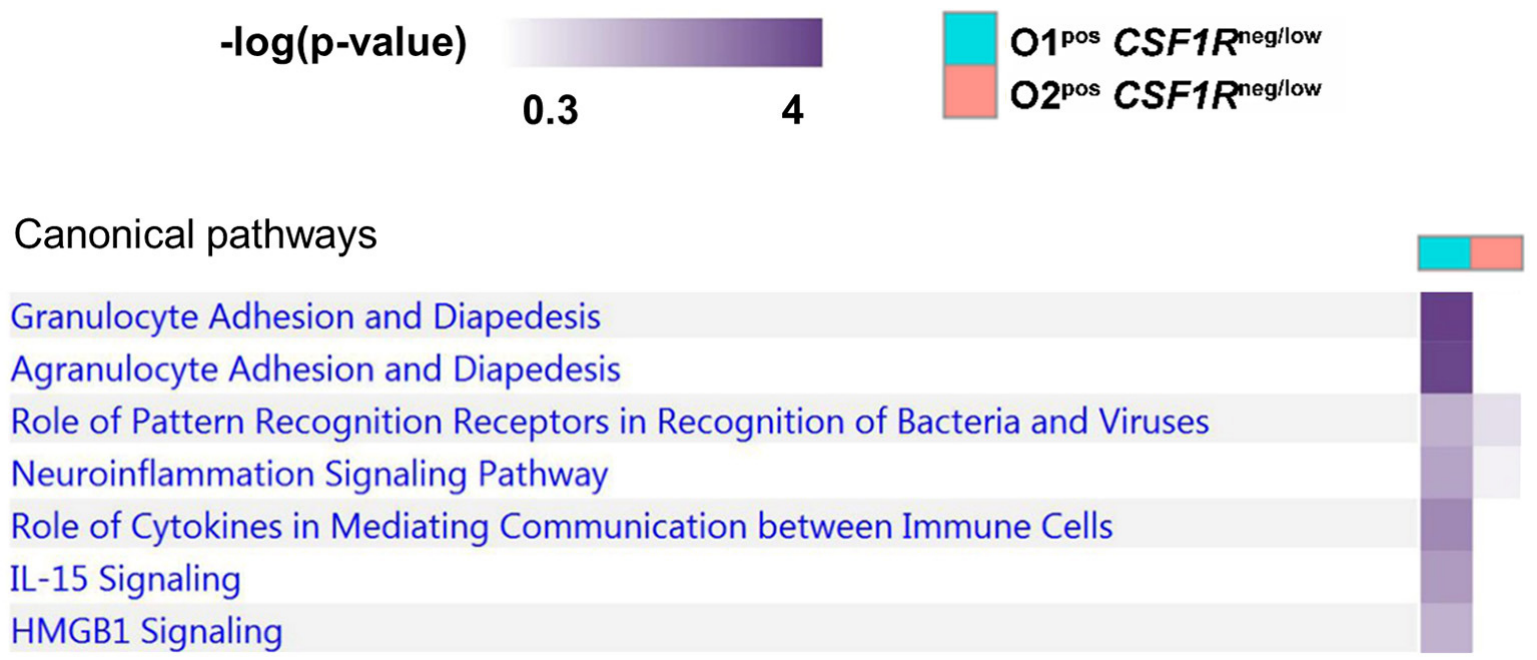
Pathway analysis of APEC O1-*GFP* vs. APEC O2-*GFP* infected *CSF1R*-tg^neg/low^ cells based on DEGs between the two populations (log fold-change). Stronger coloring indicates enrichment of genes associated with the given pathway.

To further examine the difference between APEC O1-*GFP* and APEC O2-*GFP* infected *CSF1R*-tg^neg/low^ cells, a pathway comparison was made (Figure 7). The pathways enriched in APEC O1-*GFP*^pos^ *CSF1R*-tg^neg/low^ cells included “granulocyte adhesion and diapedesis” (*CCL19, CX3CL1, CXCL8, VCAM1*), “Pattern Recognition Receptors in Recognition of Bacteria” (*CXCL8 and PTX3*), “Role of cytokines in mediating communication” (*CXCL8, IL22*), and “IL15 and HMGB1 signaling” (*CXCL8, VCAM1*). Similar to the pathways enriched in the *CSF1R*-tg^high^ infected cells, the pathways showed more enrichment in the APEC O1-*GFP* infected cells relative to the APEC O2-*GFP* infected cells.

### Bacterial Invasion Contributes to APEC Uptake *in vitro*

**Figure 8:**
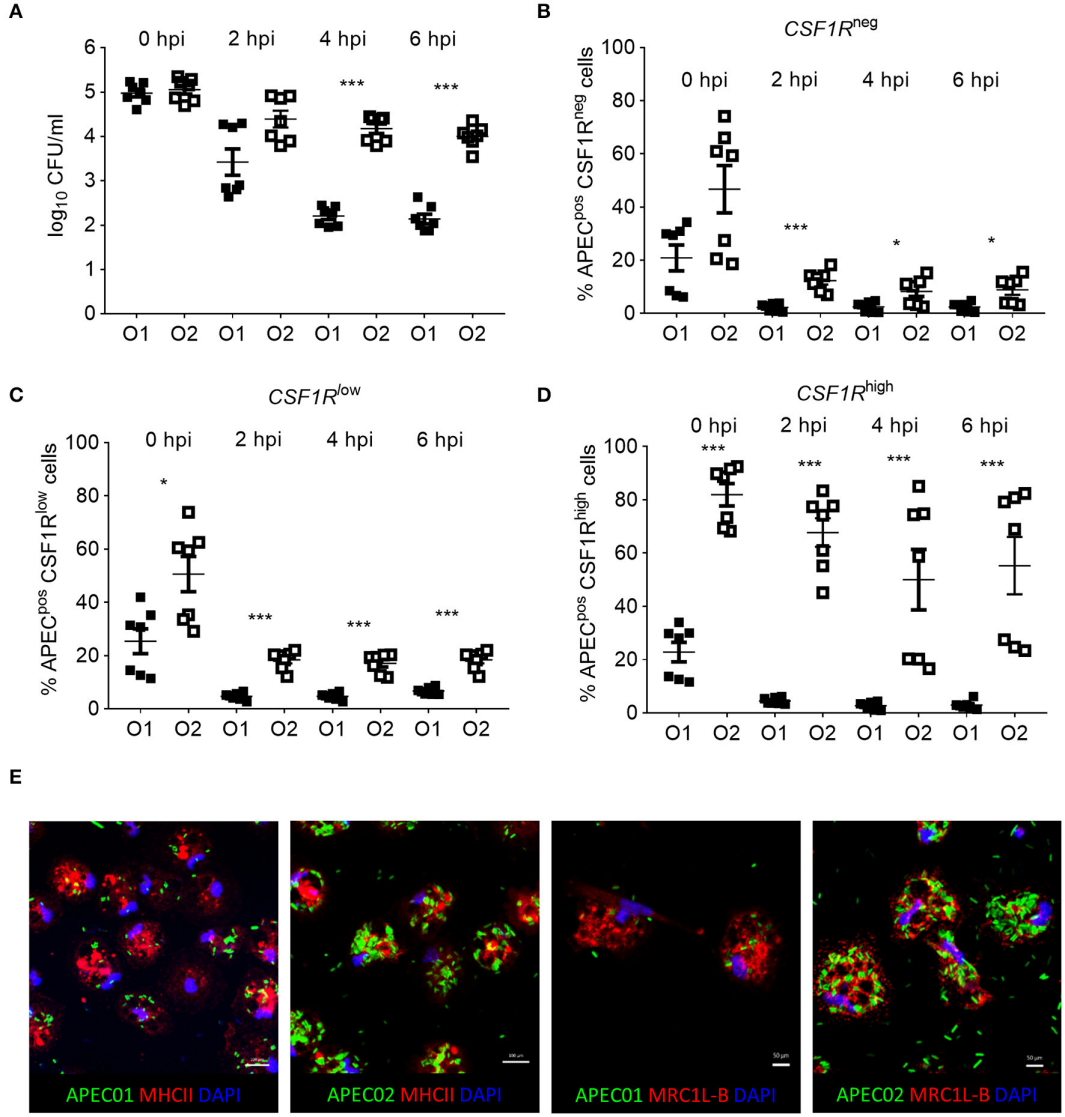
*In vitro* analysis of APEC^pos^ lung cells. **(A)** Enumeration of viable bacteria within freshly isolated gradient purified lung cells inoculated with an MOI = 10 at 0, 2, 4, and 6 hpi, **(B–D)** quantification of APEC^pos^ cells within the *CSF1R*-tg^neg^, *CSF1R*-tg^low^, and *CSF1R*-tg^high^ cells of the same samples by flow cytometry, and **(E)** visualization of APEC infection post MOI = 10 inoculation of adherent *CSF1R*-tg^high^ cells by confocal microscopy. **(A–D)** *n* = 7 birds from two independent studies, the mean with SEM is shown, ^*^P < 0.05, ^***^P < 0.001. **(E)** Data from one representative bird.

**Figure 9:**
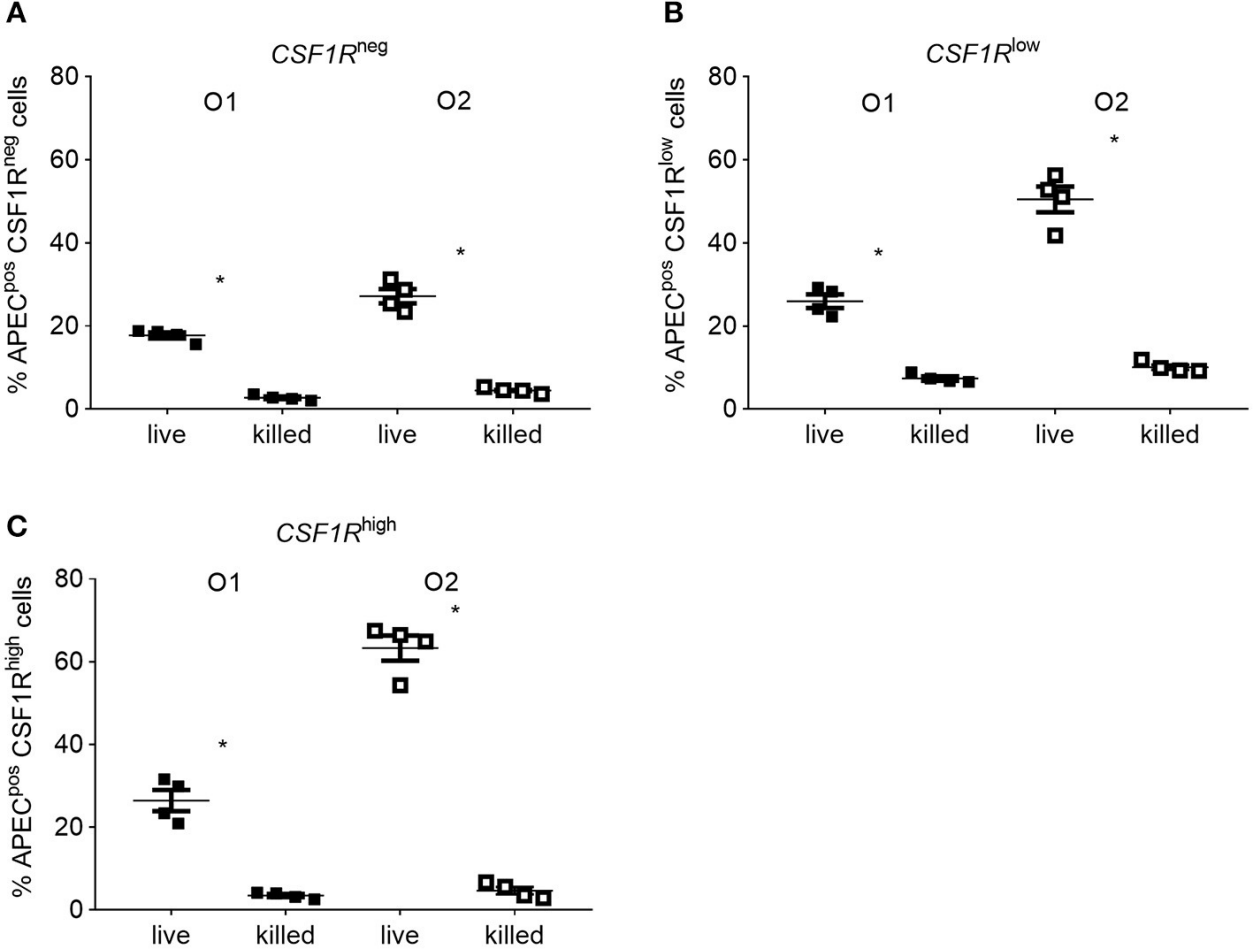
*In vitro* flow cytometric analysis of APEC^pos^ lung cells after inoculation with live or heat killed APEC. Gradient purified lung cells were isolated from 6 to 8 weeks old *CSF1R*-reporter transgenic birds and inoculated with an MOI = 10 of either live or heat killed APEC O1-*GFP* or APEC O2-*GFP*. **(A–C)** Quantification of APEC^pos^ cells in the *CSF1R*-tg^neg^, *CSF1R*-tg^low^, and *CSF1R*-tg^high^ cells. O1 is APEC O1-*GFP*, O2 is APEC O2-*GFP*. *n* = 4 birds from two independent studies. The mean with SEM is shown. ^*^P < 0.05.

*in vitro* phagocytosis and killing assays were performed using primary lung cells of *CSF1R*-reporter transgenic birds and APEC O1-*GFP* or APEC O2-*GFP* (MOI = 10), revealing differences in cell tropism and intracellular survival between the APEC strains (Figure 8). The total number of viable intracellular bacteria detected after infection and ceftazidime treatment at the “zero hpi” interval did not differ significantly between the APEC strains (Figure 8A). At this time, APEC O1-*GFP* infected around 20% of *CSF1R*-tg^neg^, *CSF1R*-tg^low^, and *CSF1R*-tg^high^ cells. In contrast, APEC O2-*GFP* infected around 80% of *CSF1R*-tg^high^ cells (Figures 8B–D). Interestingly, APEC O2-*GFP* survived longer in lung cells than APEC O1-*GFP*, with statistically significant differences in recovery of the strains from 4 hpi (Figure 8A). The percentage of APEC O2-*GFP*^pos^ *CSF1R*-tg^high^ cells did not significantly decrease post-inoculation (Figure 8D; *P* = 0.053 between 0 and 6 hpi; in addition to significant differences between the strains at each time point as indicated), whilst the percentage of APEC O1-*GFP*^pos^ *CSF1R*-tg^high^ cells significantly decreased over time (*P* = 0.0006 between 0 and 6 hpi). Confocal microscopy of adherent *CSF1R*-tg^high^ cells confirmed the higher number of APEC O2-*GFP* infected cells (Figure 8E), and that bacteria were intracellular (Supplementary Figure 2). The relative proportions of *CSF1R*-tg^neg^, *CSF1R*-tg^low^, and *CSF1R*-tg^high^ cells in the isolated cell populations used for these *in vitro* studies is shown in Supplementary Figure 3, and shows a large *CSF1R*-tg^neg^ population and small *CSF1R*-tg^low^ and *CSF1R*-tg^high^ populations as expected. This indicates that APEC O2-*GFP* were phagocytized by, or actively invaded, the *CSF1R*-tg^high^ cells as a higher percentage of *CSF1R*-tg^high^ cells were APEC O2-*GFP*^pos^ compared to the other cell populations whilst the *CSF1R*-tg^high^ cells represent the smallest population. To determine if APEC were phagocytized or actively invading the *CSF1R*-tg^high^ and *CSF1R*-tg^low^ cells, the assay was repeated with live and heat killed bacteria. Heat killed bacteria were phagocytosed at low levels, whereas up to 60% of *CSF1R*-tg^high^ and *CSF1R*-tg^low^ cells contained the live bacteria suggesting that active invasion is the primary route of entry (Figure 9).

## Discussion

In this study the interactions between *GFP*-expressing strains of APEC serotypes O1:K1:H7 and O2:K1:H5 and phagocytic cells in the lung of *CSF1R*-reporter transgenic chickens at 6 hpi were investigated. When analysing events early after inoculation it is essential to deliver consistent doses to avoid dose-related differences in innate responses. The intra-air sac route was chosen in this study as it was previously shown to produce reproducible bacterial loads chicken lungs (5, 10, 25). In our study *CSF1R*-reporter transgenic chickens were inoculated with 10^9^ CFU. While this is unlikely to reflect natural exposure, our pilot dose-titration studies indicated that this dose of APEC was a prerequisite to detect >1% APEC^pos^ leukocytes in the lung (data not shown). We were also mindful that cloning vectors and fluorescent proteins have been reported to attenuate bacterial pathogens, including pFVP25.1 used in this study and which had a modest impact on *Salmonella* invasion *in vitro* (26). However, no impact of pFVP25.1 on APEC invasion or *in vitro* growth was detected (data not shown) and we observed that the APEC O1-*GFP* and APEC O2-*GFP* strains rapidly induced pathology similar to that of the parent strains and that they were capable of efficient systemic translocation.

Flow cytometry analysis of lung cells from *CSF1R*-trangenic chickens indicate the presence of three cell phenotypes based on transgene expression, *CSF1R*-tg^high^, *CSF1R*-tg^low^, and *CSF1R*-tg^neg^. In this study the *CSF1R*-tg^neg/low^ cells were identified as the main cell population associated with APEC O1-*GFP* or APEC O2-*GFP in vivo* and flow cytometric and transcriptomic analysis identified them as heterophils, the chicken counterpart of mammalian neutrophils. Significantly lower numbers of bacteria were associated with *CSF1R*-tg^high^ cells, identified as macrophages and DCs. Compared to *CSF1R*-tg^high^ cells from uninfected birds many DC related genes were not upregulated in APEC-infected birds (*FLT3, CADM1, CIITA, CCR7, XCR1*), indicating that APEC^pos^ *CSF1R*-tg^high^ cells were enriched for macrophages, likely recruited from the circulation due to strong inflammatory signals produced by epithelial cells and resident innate cells in the lung to limit the infection. These data indicate an important role for the early cellular innate immune response in the control of colibacillosis, driven primarily by heterophils and to a lesser extent by macrophages, both cells known to be important in bacterial clearance and immunomodulation.

Low percentages of APEC^pos^ *CSF1R*-tg^neg/low^ and *CSF1R*-tg^high^ cells after APEC O1-*GFP* or APEC O2-*GFP* inoculation may be explained by the possible ability of APEC to evade phagocytosis due to the expression of surface polysaccharides, fimbriae and other APEC virulence factors. In particular, the *E. coli* K1 polysialic acid capsule, expressed by both APEC strains selected for this study, is hypothesized to contribute toward the evasion of phagocytosis (6), although *in vivo* data confirming this are lacking. Our *in vivo* data show the percentage of APEC uptake by *CSF1R*-tg^low^ and *CSF1R*-tg^high^ cells was similar to those observed after *in vitro* inoculation of lung leukocytes with heat killed APEC. However, the percentage of APEC uptake *in vitro* using live bacteria was significantly higher and APEC O2-*GFP* seem to preferentially be taken up by the *CSF1R*-tg^high^ cells. This demonstrates a clear discrepancy between APEC uptake *in vivo* and *in vitro*, and studies that predict the outcome of APEC infections in poultry from *in vitro* data alone must therefore be interpreted with caution.

In our study the response of heterophils, macrophages and DCs to APEC O1-*GFP* and O2-*GFP* infection in birds was overall similar. This response involved inflammatory pathways including IL-8, IL-6 and Th_1_ pathway signaling, suggesting a robust innate immune response at 6 hpi. Previous transcriptome studies analysing bone marrow, thymus, bursa, spleen and blood derived leukocytes, in birds with differing susceptibility to APEC O1:K1:H7 infection, also revealed an important role for the innate immune response (25, 27). At 1 dpi differences in β-defensins, *CD74*, and *IL8* expression between challenged and control birds were observed. However, in contrast to our study these studies did not analyse lung tissue or individual immune cells and focused on the adaptive immune response at later time points post 10^8^ CFU APEC O1. A direct comparison was therefore not feasible, and we chose a higher dose of inoculation which seemed ideal to induce a strong cellular innate immune response 6 hpi based on our pilot data. Strikingly in our study investigating the very early cellular innate immune response, many pathways were more highly repressed or less activated in APEC O2-*GFP* inoculated birds compared to APEC O1-*GFP*, in both heterophils, macrophages and DCs. Most notably pathways associated with the Aryl Hydrocarbon Receptor (AhR), an important emerging modulator of inflammatory signaling, IL-17 and STAT3 associated signaling were more highly repressed or less activated during APEC O2-*GFP* infection.

*AhR* is a ligand-dependent transcription factor that not only senses environmental toxins, but also bacterial virulence factors. In mice it was shown to be an intracellular pattern recognition receptor that regulates immune and degradation pathways in myeloid and epithelial cells (28). *AhR* is evolutionarily conserved amongst many species including birds (29, 30). AhR sensing leads to transcription of canonical detoxifying genes of which *CYP1A1* and *CYP1A2* are key members and in our study amongst the most repressed genes post-APEC O2-*GFP* inoculation compared to APEC O1-*GFP* (Figure 6). In addition, AhR regulates cytokine and chemokine production, inflammatory leukocyte recruitment and control of bacterial replication and LPS-induced inflammatory responses (28, 31). Inflammatory pathways such as Th_1_, NF-κB, and STAT3 pathways and their associated cytokines were generally more highly repressed or less activated post-APEC O2-*GFP* inoculation compared to APEC O1-*GFP* in our study. The pro-inflammatory cytokines IL-6 and IL-1β were strongly induced post-APEC O1-*GFP* and slightly higher post-APEC O2-*GFP* infection in macrophages.

In our study, the immunomodulatory cytokine genes *IL17* and *IL22* were less upregulated in APEC O2-*GFP* infected birds' macrophages and heterophils, respectively, whereas *IL-10* was upregulated in macrophages to the same extent after APEC O1-*GFP* and O2-*GFP* inoculation. In mammalian studies with LPS-stimulated murine peritoneal macrophages, loss of *AhR* resulted in inhibited IL-10 production (31, 32), and attenuated IL-17 and no IL-22 production was linked to impairment of the Th_17_ response (33). We observed significantly less pathology in APEC O2-*GFP* infected birds whilst the bacterial load was similar to APEC O1-*GFP*, possibly a consequence of dampened IL-17 related signaling. In addition, the induction of systemic responses via the acute-phase response and increasing serum concentrations of CSF3 and CSF2 is linked to an increase in neutrophil and macrophage numbers at the site of infection in mice, and is regulated by IL-17 amongst other cytokines (34). This observation merits further study over a wider series of sampling intervals. It should also be appreciated that differences at transcript level cannot be taken to mean differences in the abundance or processing of individual proteins, which require further validation prior to in-depth follow-on studies. Overall it draws a complex picture of *AhR* induced signaling in cellular innate immunity in response to bacterial challenge and indicates that after APEC inoculation, *AhR* plays a complex role in the pro- and anti-inflammatory cytokine production and its effect on the immune system is modulated by the APEC strain.

Notably in our data, *PTX3* was less expressed in APEC O2-*GFP* infected heterophils compared to APEC O1-*GFP* infected cells. *PTX3* is rapidly produced and released by mononuclear phagocytes and DCs in response to inflammatory signals (35). In chickens, it was shown to be strongly upregulated post-APEC O1:K1:H7 inoculation (36). Recent findings in mammals have shown that pentraxins are involved in complement activation and amplification via communication with complement initiation pattern recognition molecules, but also via recruitment of complement regulators (37). In our data, the complement component, *C4A* and complement regulator, *CSMD2* were downregulated in APEC O2-*GFP* infected macrophages and DCs compared to APEC O1-*GFP*. A role for PTX3 in the complement-mediated clearance of apoptotic cells was also suggested where it limits C1q-mediated complement activation and binds to apoptotic cells and inhibits their clearance by DCs (38, 39).

In our data, *CASP7* and the chicken homolog of *ZNF420* were less expressed in APEC O2-*GFP* infected macrophages and DCs, and *DTHD1* and *EVA1A* were less expressed in APEC O2-*GFP* infected heterophils. All these genes are important in the apoptotic response. Other genes and pathways less expressed in APEC O2-*GFP* infected heterophils included the granulocyte adhesion and diapedesis pathway, and particularly *CCL19, CX3CL1, CXCL8*, and *VCAM1* expression, with important roles in attracting heterophils to the site of infection and promoting adhesin to endothelial cells. Overall, lower expression of *IL17, IL22, PTX3*, and heterophil attracting cytokines, such as *CXCL8* and *VCAM1* in our data suggests a dampened innate immune response post APEC O2-*GFP* infection. This could at least partially explain the higher bacterial loads observed in the left lung, opposite the inoculation site, and liver post-APEC O2-*GFP* inoculation.

In conclusion, our data identified heterophils and macrophages as the main APEC-bearing lung phagocytes post-APEC O1-*GFP* or APEC O2-*GFP* inoculation of chickens, in an innate immune response dominated by heterophils. Transcriptomic analysis identified many inflammatory pathways including IL-8, IL-6, and T_h_1 were strongly upregulated post-APEC O1-*GFP* or APEC O2-*GFP* inoculation. Strikingly our analysis revealed many pathways and genes, particularly related to *AhR, IL17* and *STAT3* signaling, heterophil recruitment and the acute phase response, were more highly repressed or less activated in APEC O2-*GFP* inoculated birds compared to APEC O1-*GFP*. Given the huge genetic diversity of APEC, observations made in this study stress the potential for observed responses to be specific to the host-pathogen combination under study. Moreover, genetic differences can also occur within an APEC serotype (40), and differences in the clinical outcome of disease after inoculation with different APEC strains of the same serotype were shown (41). Thus, it will be important not to assume that responses detected with single strains will necessarily apply to other strains of a different or the same serotype. In primary lung leukocytes *in vitro*, APEC O2-*GFP* exhibited higher net intracellular survival over time compared to APEC O1-*GFP*. However, we observed a clear discrepancy between APEC uptake *in vivo* and *in vitro*. Our data revealed important differences in the cellular innate immune response between APEC strains O1:K1:H7 and O2:K1:H5 and suggest that APEC O2-*GFP* may subvert or evade immune responses in phagocytic cells to a greater extent than APEC O1-*GFP*. Future experiments will focus on the functional consequences of the APEC-cell interactions and include time series leading to resolution or distinct pathologies.

## Data Availability Statement

The datasets generated and analyzed for this study are included in the published article (and its additional files) or in the following data repository; RNA-seq data has been submitted to the European Nucleotide Archive (PRJEB35225) (https://www.ebi.ac.uk/ena/data/view/PRJEB35225).

## Ethics Statement

The animal study was reviewed and approved by the Animal Welfare and Ethical Review Board of The Moredun Research Institute, Scotland, United Kingdom. Animals were bred and housed in premises licensed under UK Home Office Establishment Licenses (PEL X212DDDBD and XA40CEF03) in full compliance with the requirements of the Animals (Scientific Procedures) Act 1986. Procedures were conducted under project license PPL 70/7860, with *CSF1R*-reporter transgenic birds bred under PPL 60/4253, with the consent of the Animal Welfare and Ethical Review Board of The Moredun Research Institute.

## Author Contributions

AA, LV, and MS conceptualized the study. AA, KB, CC-U, DB, and LV performed or assisted with the animal experiments. KM and MM performed the transcriptomic data analysis. KS performed the confocal staining and analysis. PK, MS, LV, CS, and SL secured the funding. CS provided resources. AA, KM, and LV wrote the manuscript. All authors contributed to, read and approved the final manuscript.

### Conflict of Interest

The authors declare that the research was conducted in the absence of any commercial or financial relationships that could be construed as a potential conflict of interest.

## References

[1] Guabiraba R Schouler C. Avian colibacillosis: still many black holes. FEMS Microbiol Lett. (2015) 362:fnv118. 10.1093/femsle/fnv118 26204893

[2] Collingwood C Kemmett K Williams N Wigley P. Is the concept of avian pathogenic *Escherichia coli* as a single pathotype fundamentally flawed? Front Vet Sci. (2014) 1:5. 10.3389/fvets.2014.00005 26664913 PMC4668852

[3] Cuperus T Coorens M van DA Haagsman HP. Avian host defense peptides. Dev Comp Immunol. (2013) 41:352–69. 10.1016/j.dci.2013.04.019 23644014

[4] Wigley P. Immunity to bacterial infection in the chicken. Dev Comp Immunol. (2013) 41:413–7. 10.1016/j.dci.2013.04.008 23648643

[5] Pourbakhsh SA Boulianne M Martineau-Doize B Dozois CM Desautels C Fairbrother JM Dynamics of *Escherichia coli* infection in experimentally inoculated chickens. Avian Dis. (1997) 41:221–33. 10.2307/1592463 9087340

[6] Mellata M Dho-Moulin M Dozois CM Curtiss R III Lehoux B Fairbrother JM. Role of avian pathogenic *Escherichia coli* virulence factors in bacterial interaction with chicken heterophils and macrophages. Infect Immun. (2003) 71:494–503. 10.1128/IAI.71.1.494-503.2003 12496200 PMC143357

[7] Ariaans MP Matthijs MG van HD van de Haar P van Eck JH Hensen EJ. The role of phagocytic cells in enhanced susceptibility of broilers to colibacillosis after Infectious Bronchitis Virus infection. Vet Immunol Immunopathol. (2008) 123:240–50. 10.1016/j.vetimm.2008.02.003 18359518 PMC7112703

[8] Horn F Correa AM Barbieri NL Glodde S Weyrauch KD Kaspers B. Infections with avian pathogenic and fecal *Escherichia coli* strains display similar lung histopathology and macrophage apoptosis. PLoS ONE. (2012) 7:e41031. 10.1371/journal.pone.0041031 22848424 PMC3405075

[9] Sutton K Costa T Alber A Bryson K Borowska D Balic A. Visualisation and characterisation of mononuclear phagocytes in the chicken respiratory tract using *CSF1R*-transgenic chickens. Vet Res. (2018) 49:104. 10.1186/s13567-018-0598-7 30305141 PMC6389226

[10] Alber A Costa T Chintoan-Uta C Bryson KJ Kaiser P Stevens MP. Dose-dependent differential resistance of inbred chicken lines to avian pathogenic *Escherichia coli* challenge. Avian Pathol. (2019) 48:157–67. 10.1080/03079457.2018.1562154 30570345

[11] Dziva F Stevens MP. Colibacillosis in poultry: unravelling the molecular basis of virulence of avian pathogenic *Escherichia coli* in their natural hosts. Avian Pathol. (2008) 37:355–66. 10.1080/03079450802216652 18622850

[12] Balic A Garcia-Morales C Vervelde L Gilhooley H Sherman A Garceau V. Visualisation of chicken macrophages using transgenic reporter genes: insights into the development of the avian macrophage lineage. Development. (2014) 141:3255–65. 10.1242/dev.105593 25063453 PMC4197536

[13] Johnson TJ Kariyawasam S Wannemuehler Y Mangiamele P Johnson SJ Doetkott C. The genome sequence of avian pathogenic *Escherichia coli* strain O1:K1:H7 shares strong similarities with human extraintestinal pathogenic *E. coli* genomes. J Bacteriol. (2007) 189:3228–36. 10.1128/JB.01726-06 17293413 PMC1855855

[14] Valdivia RH Falkow S. Bacterial genetics by flow cytometry: rapid isolation of *Salmonella typhimurium* acid-inducible promoters by differential fluorescence induction. Mol Microbiol. (1996) 22:367–78. 10.1046/j.1365-2958.1996.00120.x 8930920

[15] Dho M Lafont JP. *Escherichia coli* colonization of the trachea in poultry: comparison of virulent and avirulent strains in gnotoxenic chickens. Avian Dis. (1982) 26:787–97. 10.2307/1589865 6760850

[16] Chanteloup NK Porcheron G Delaleu B Germon P Schouler C Moulin-Schouleur M. The extra-intestinal avian pathogenic *Escherichia coli* strain BEN2908 invades avian and human epithelial cells and survives intracellularly. Vet Microbiol. (2011) 147:435–9. 10.1016/j.vetmic.2010.07.013 20708353

[17] Bolger AM Lohse M Usadel B. Trimmomatic: a flexible trimmer for Illumina sequence data. Bioinformatics. (2014) 30:2114–20. 10.1093/bioinformatics/btu170 24695404 PMC4103590

[18] Dobin A Davis CA Schlesinger F Drenkow J Zaleski C Jha S. STAR: ultrafast universal RNA-seq aligner. Bioinformatics. (2013) 29:15–21. 10.1093/bioinformatics/bts635 23104886 PMC3530905

[19] Liao Y Smyth GK Shi W. featureCounts: an efficient general purpose program for assigning sequence reads to genomic features. Bioinformatics. (2014) 30:923–30. 10.1201/b16589 24227677

[20] Robinson MD McCarthy DJ Smyth GK. edgeR: a Bioconductor package for differential expression analysis of digital gene expression data. Bioinformatics. (2010) 26:139–40. 10.1093/bioinformatics/btp616 19910308 PMC2796818

[21] Thomas PD Campbell MJ Kejariwal A Mi H Karlak B Daverman R. PANTHER: a library of protein families and subfamilies indexed by function. Genome Res. (2003) 13:2129–41. 10.1101/gr.772403 12952881 PMC403709

[22] Theocharidis A van DS Enright AJ Freeman TC. Network visualization and analysis of gene expression data using BioLayout Express(3D). Nat Protoc. (2009) 4:1535–50. 10.1038/nprot.2009.177 19798086

[23] Garrido D Alber A Kut E Chanteloup NK Lion A Trotereau A. The role of type I interferons (IFNs) in the regulation of chicken macrophage inflammatory response to bacterial challenge. Dev Comp Immunol. (2018) 86:156–70. 10.1016/j.dci.2018.04.025 29729283

[24] Sekelova Z Stepanova H Polansky O Varmuzova K Faldynova M Fedr R. Differential protein expression in chicken macrophages and heterophils *in vivo* following infection with *Salmonella Enteritidis*. Vet Res. (2017) 48:35. 10.1186/s13567-017-0439-0 28623956 PMC5473982

[25] Sandford EE Orr M Shelby M Li X Zhou H Johnson TJ. Leukocyte transcriptome from chickens infected with avian pathogenic *Escherichia coli* identifies pathways associated with resistance. Results Immunol. (2012) 2:44–53. 10.1016/j.rinim.2012.02.003 24371566 PMC3862389

[26] Knodler LA Bestor A Ma C Hansen-Wester I Hensel M Vallance BA. Cloning vectors and fluorescent proteins can significantly inhibit *Salmonella enterica* virulence in both epithelial cells and macrophages: implications for bacterial pathogenesis studies. Infect Immun. (2005) 73:7027–31. 10.1128/IAI.73.10.7027-7031.2005 16177386 PMC1230934

[27] Sun H Bi R Liu P Nolan LK Lamont SJ. Combined analysis of primary lymphoid tissues' transcriptomic response to extra-intestinal *Escherichia coli* (ExPEC) infection. Dev Comp Immunol. (2016) 57:99–106. 10.1016/j.dci.2015.12.013 26710679

[28] Moura-Alves P Fae K Houthuys E Dorhoi A Kreuchwig A Furkert J. AhR sensing of bacterial pigments regulates antibacterial defence. Nature. (2014) 512:387–92. 10.1038/nature13684 25119038

[29] Kennedy SW Lorenzen A Jones SP Hahn ME Stegeman JJ. Cytochrome P4501A induction in avian hepatocyte cultures: a promising approach for predicting the sensitivity of avian species to toxic effects of halogenated aromatic hydrocarbons. Toxicol Appl Pharmacol. (1996) 141:214–30. 10.1016/S0041-008X(96)80027-5 8917694

[30] Hahn ME Karchner SI Shapiro MA Perera SA. Molecular evolution of two vertebrate aryl hydrocarbon (dioxin) receptors (AHR1 and AHR2) and the PAS family. Proc Natl Acad Sci USA. (1997) 94:13743–8. 10.1073/pnas.94.25.13743 9391097 PMC28377

[31] Kimura A Naka T Nakahama T Chinen I Masuda K Nohara K. Aryl hydrocarbon receptor in combination with Stat1 regulates LPS-induced inflammatory responses. J Exp Med. (2009) 206:2027–35. 10.1084/jem.20090560 19703987 PMC2737163

[32] Zhu J Luo L Tian L Yin S Ma X Cheng S. Aryl hydrocarbon receptor promotes IL-10 expression in inflammatory macrophages through Src-STAT3 signaling pathway. Front Immunol. (2018) 9:2033. 10.3389/fimmu.2018.02033 30283437 PMC6156150

[33] Veldhoen M Hirota K Christensen J O'Garra A Stockinger B. Natural agonists for aryl hydrocarbon receptor in culture medium are essential for optimal differentiation of Th17 T cells. J Exp Med. (2009) 206:43–9. 10.1084/jem.20081438 19114668 PMC2626686

[34] Ye P Rodriguez FH Kanaly S Stocking KL Schurr J Schwarzenberger P. Requirement of interleukin 17 receptor signaling for lung CXC chemokine and granulocyte colony-stimulating factor expression, neutrophil recruitment, and host defense. J Exp Med. (2001) 194:519–27. 10.1084/jem.194.4.519 11514607 PMC2193502

[35] Doni A Peri G Chieppa M Allavena P Pasqualini F Vago L Production of the soluble pattern recognition receptor PTX3 by myeloid, but not plasmacytoid, dendritic cells. Eur J Immunol. (2003) 33:2886–93. 10.1002/eji.200324390 14515272

[36] Burkhardt NB Roll S Staudt A Elleder D Hartle S Costa T. The long pentraxin PTX3 is of major importance among acute phase proteins in chickens. Front Immunol. (2019) 10:124. 10.3389/fimmu.2019.00124 30774632 PMC6367253

[37] Ma YJ Garred P. Pentraxins in complement activation and regulation. Front Immunol. (2018) 9:3046. 10.3389/fimmu.2018.03046 30619374 PMC6305747

[38] Baruah P Dumitriu IE Peri G Russo V Mantovani A Manfredi AA. The tissue pentraxin PTX3 limits C1q-mediated complement activation and phagocytosis of apoptotic cells by dendritic cells. J Leukoc Biol. (2006) 80:87–95. 10.1189/jlb.0805445 16617159

[39] Rovere P Peri G Fazzini F Bottazzi B Doni A Bondanza A. The long pentraxin PTX3 binds to apoptotic cells and regulates their clearance by antigen-presenting dendritic cells. Blood. (2000) 96:4300–6. 10.1182/blood.V96.13.4300 11110705

[40] Dziva F Hauser H Connor TR van Diemen PM Prescott G Langridge GC. Sequencing and functional annotation of avian pathogenic *Escherichia coli* serogroup O78 strains reveal the evolution of *E. coli* lineages pathogenic for poultry via distinct mechanisms. Infect Immun. (2013) 81:838–49. 10.1128/IAI.00585-12 23275093 PMC3584874

[41] Pourbakhsh SA Boulianne M Martineau-Doize B Fairbrother JM. Virulence mechanisms of avian fimbriated *Escherichia coli* in experimentally inoculated chickens. Vet Microbiol. (1997) 58:195–213. 10.1016/S0378-1135(97)00163-6 9453131

