# Supplementary material for: Avian Pathogenic Escherichia coli (APEC) Strain-Dependent Immunomodulation of Respiratory Granulocytes and Mononuclear Phagocytes in CSF1R-Reporter Transgenic Chickens
Source: Front Immunol. 2020 Jan 10;10:3055. doi: 10.3389/fimmu.2019.03055 (PMC6967599; doi:10.3389/fimmu.2019.03055)
Supplement: Supplementary file 1 [file Data_Sheet_1.DOCX]

Supplementary Material

**Supplementary Figure 1**

Phenotyping of lung cells infected with APEC by flow cytometry. Gradient purified lung cells were isolated and subjected to flow cytometric analysis to phenotype APEC^pos^ cells against a panel of cell surface markers. (A) Representative graphs of APEC O1-*GFP* inoculated birds gated for single, live cells. APEC O1-*GFP*^pos^ cells were gated against *CSF1R-*transgene in the top left graph visualising the APEC O1-*GFP*^pos^ cells amongst the *CSF1R*-tg^neg^, *CSF1R*-tg^low^ and *CSF1R*-tg^high^ cells. The horizontal gate divides the *CSF1R*-tg^neg^ and *CSF1R*-tg^low^ cells. Further phenotypic analysis showed that APEC O1-*GFP*^pos^ cells are CD45^pos^ CD11^pos^ GRL1^pos^ GRL2^pos^, and CD3^neg^ chB6^neg^ K1^neg/low^. A positive and negative MRC1L-B and MHC II cell population was detected within the APEC O1-*GFP*^pos^ cells and these were thus further analysed in (B) showing that *CSF1R*-tg^high^ but not *CSF1R*-tg^low^ cells are MRC1L-B^pos^ and MHC II^pos^. (C) Representative graphs of APEC O2-*GFP* inoculated birds gated for single live cells and plotted identically to the APEC O1-*GFP* inoculated birds without the GRL1 and GRL2 antibody staining which was only performed with APEC O1-*GFP* inoculated birds. APEC O2-*GFP*^pos^ cells were CD45^pos^ CD11^pos^, and CD3^neg^ chB6^neg^ K1^neg/low^. Again, a positive and negative MRC1L-B and MHC II cell population was detected within the APEC O2-*GFP^pos^* cells and these were thus further analysed in (D) showing that *CSF1R*-tg^high^ but not *CSF1R*-tg^low^ cells are MRC1L-B^pos^ and MHC II^pos^. (E) Gating strategy applied to all samples and (F) analysis of ungated single cells to show that APEC^pos^ cells were not out-gated by P1 gating and that APEC^pos^ cells were mostly alive 6 hpi. Highly similar results were obtained for all APEC O1-*GFP* and APEC O2-*GFP* inoculated birds.


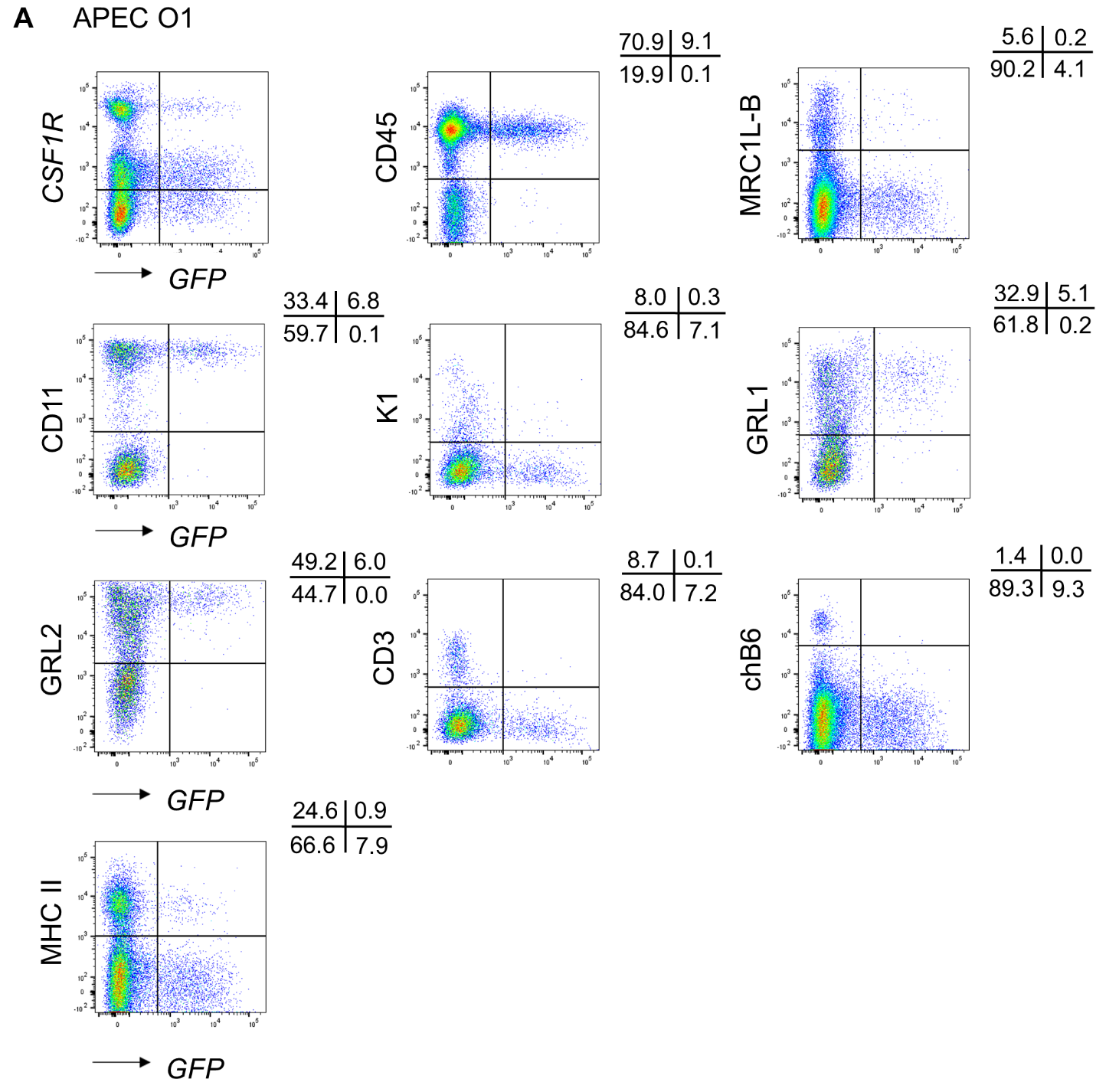

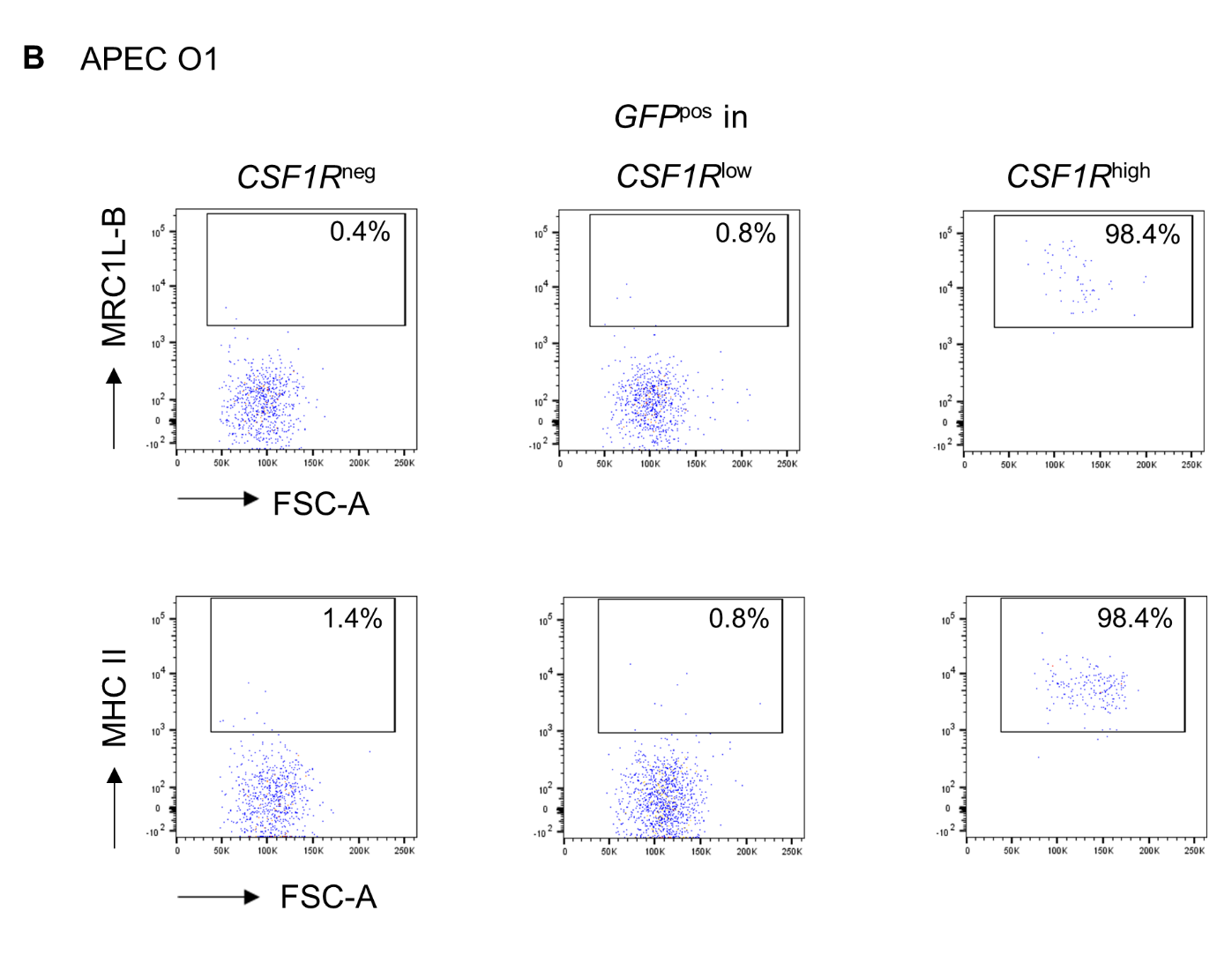

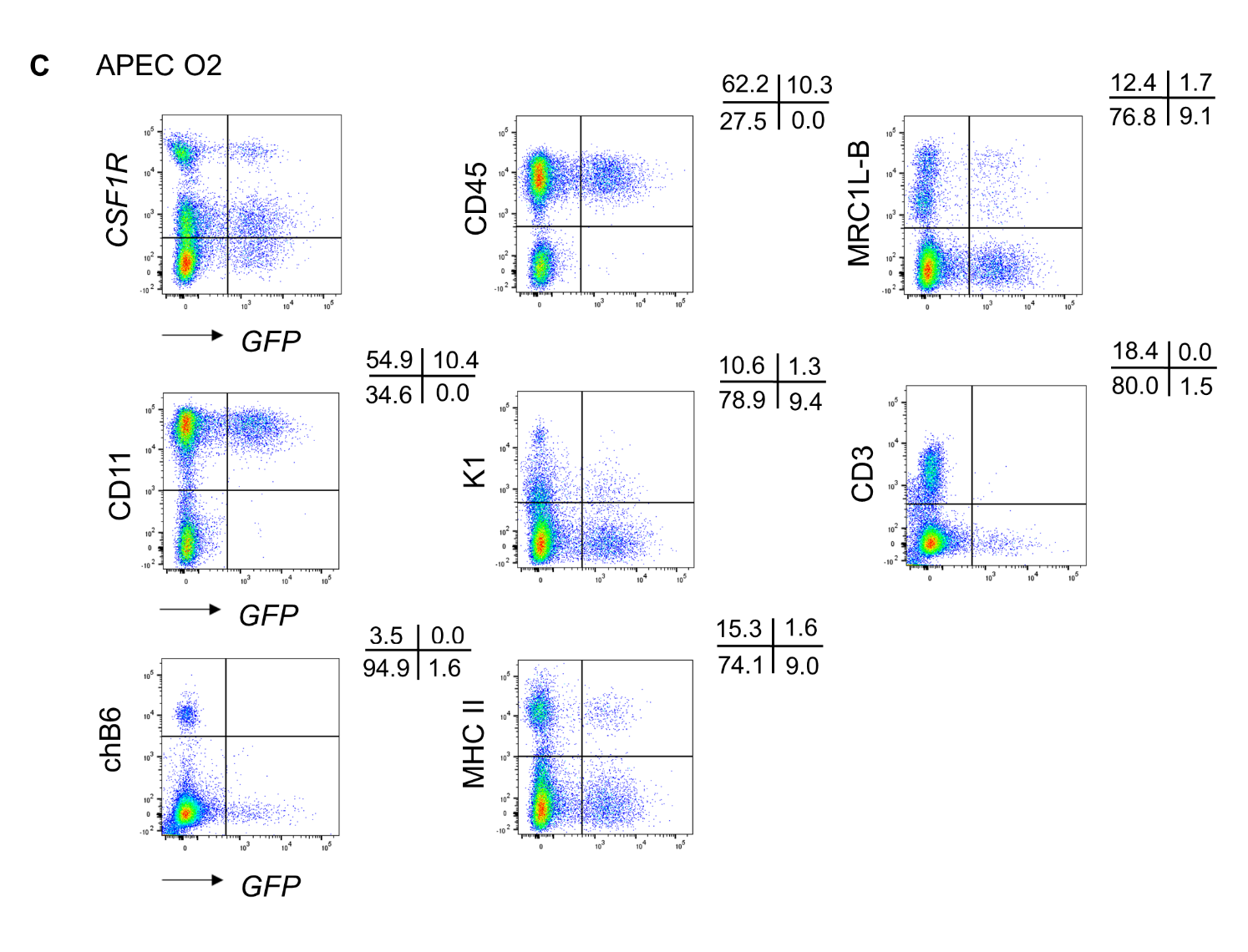

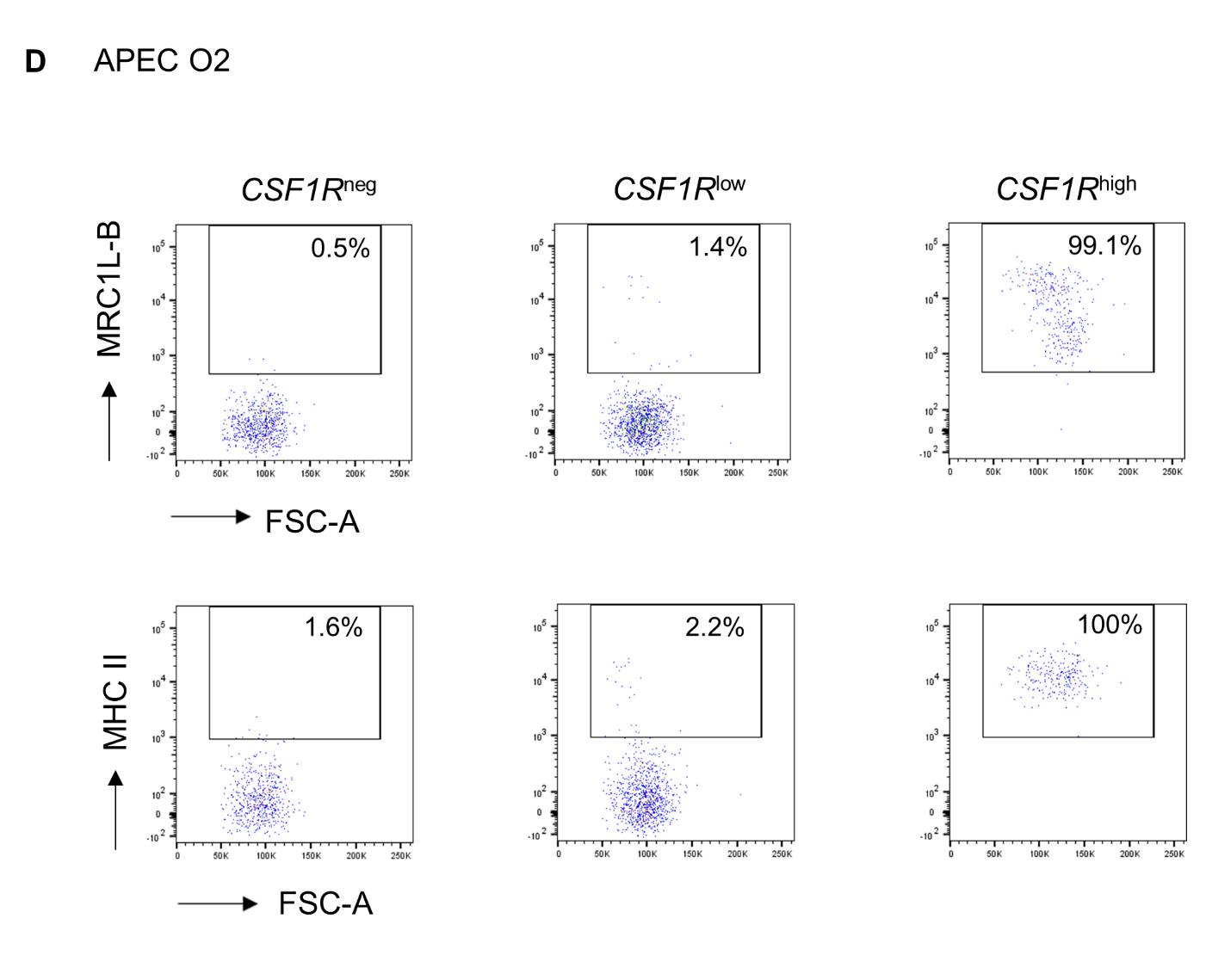

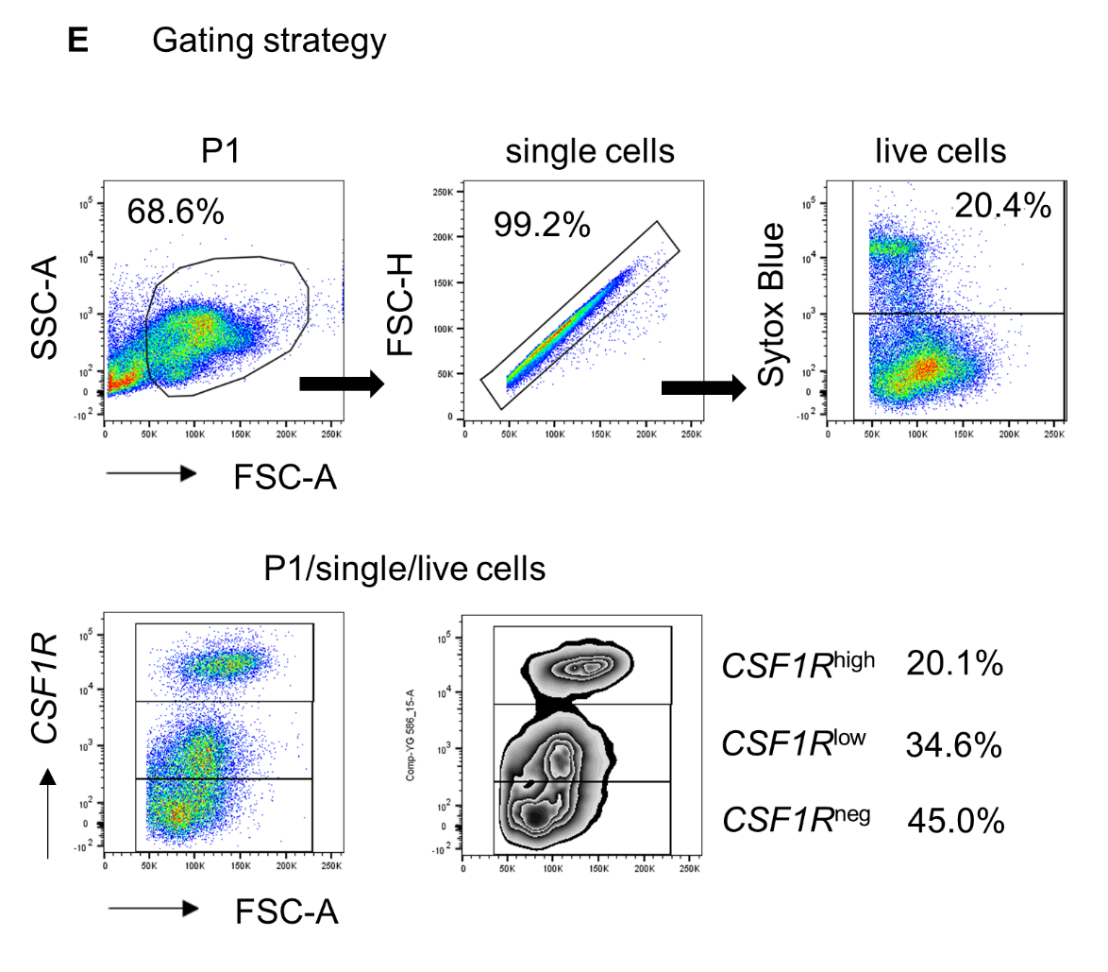

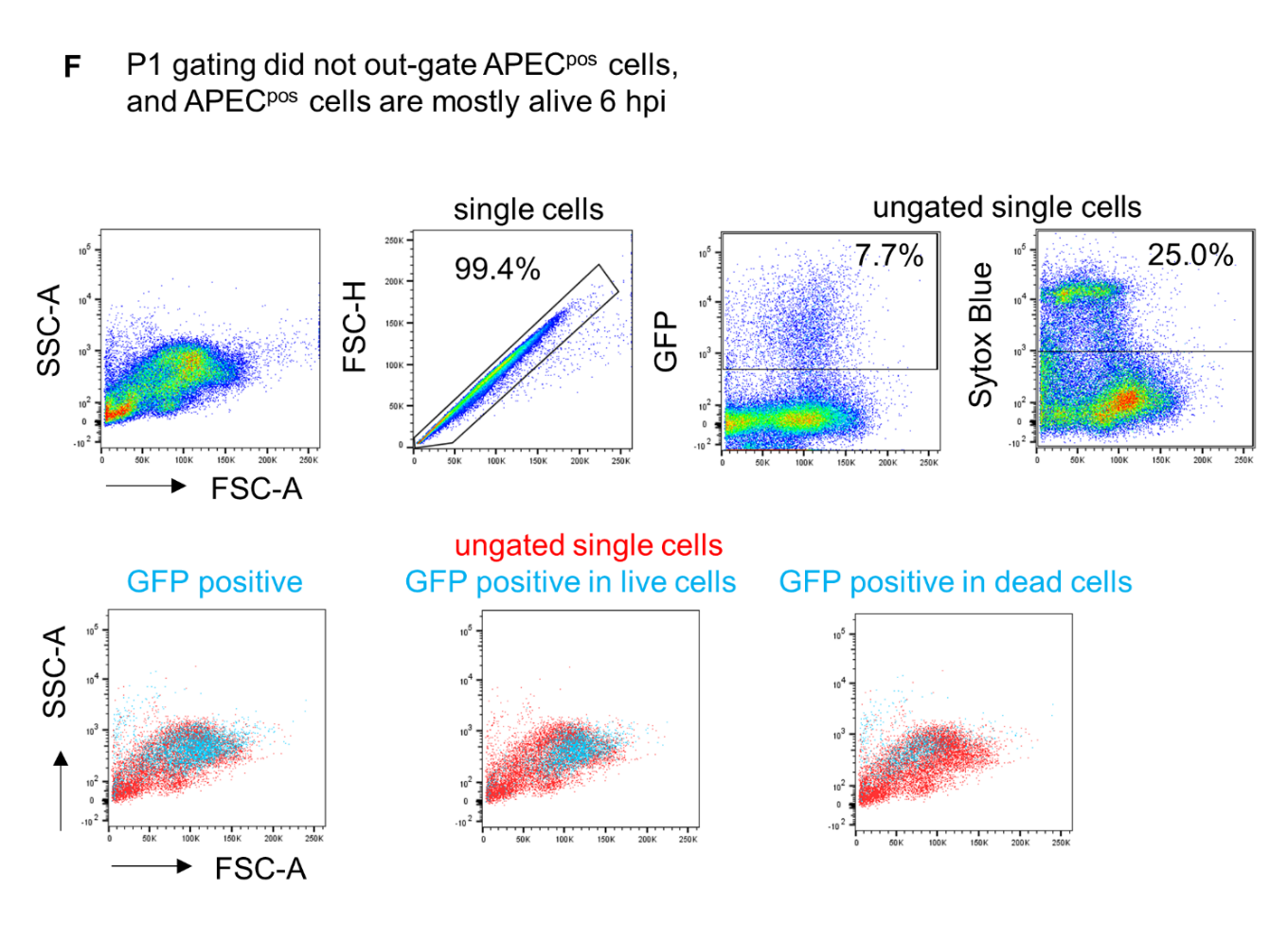


**Supplementary Figure 2**

Localisation of APEC O1-*GFP* and APEC O2-*GFP* in adherent *CSF1R*-tg^high^ lung cells by performing confocal microscopy and Z-stacks. Data derive from a representative experiment.


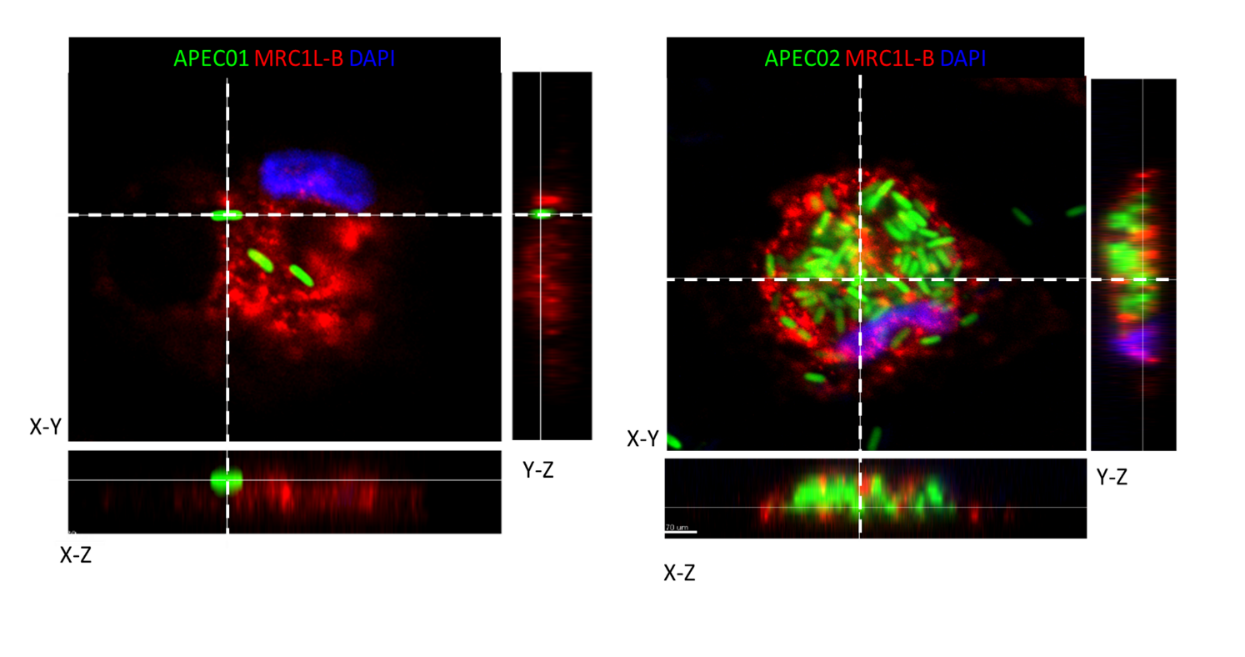


**Supplementary Figure 3**

*In vitro* analysis of *CSF1R*-tg^pos^ lung cells. Quantification of *CSF1R*-tg^neg^, *CSF1R*-tg^low^ and *CSF1R*-tg^high^ cells as percentage of single viable cells by flow cytometry, visualising the ratio between these cell populations analysed in Figure 8. *n* = 7 birds from two independent studies.


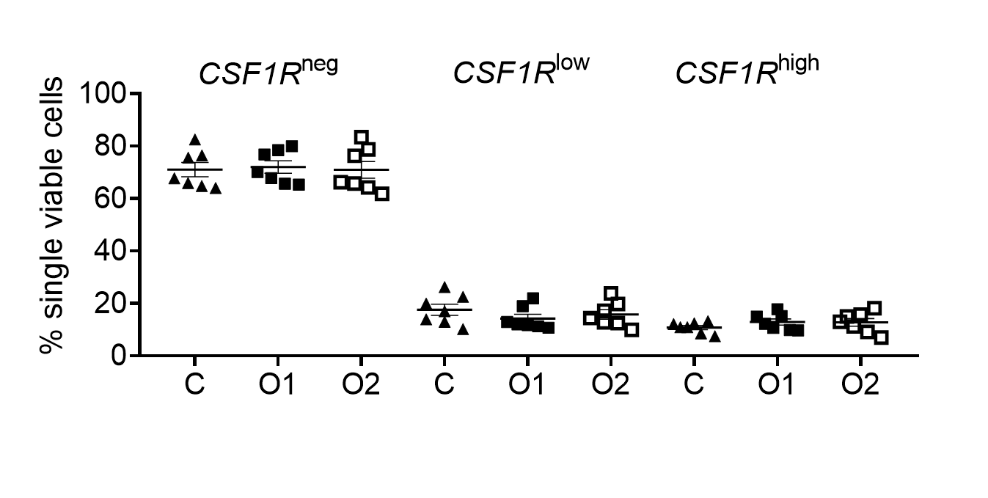


**Supplementary Table 1**

Sample overview of the RNA samples prepared for transcriptomic analysis of the APEC O1-*GFP* inoculated birds.

|  |  | APEC O1-*GFP* | |
| --- | --- | --- | --- |
| *CSF1R*^high^ PBS | *CSF1R*^high^ O1^neg^ | *CSF1R*^neg/low^ O1^pos^ | *CSF1R*^high^ O1^pos^ |
| B1 | B7 | B7 | B5 B6 |
| B2 | B8 | B8 | B7 B20 |
| B3 | B9 | B9 | B9 B19 |
| B13 | B10 | B10 | B10 B8 |
| B15 | B19 | B19 |  |
| B17 | B20 |  |  |
| *n* = 6 | *n* = 6 | *n* = 5 | *n* = 4 |

**Supplementary Table 2**

Sample overview of the RNA samples prepared for transcriptomic analysis of the APEC O2-*GFP* inoculated birds.

|  |  | APEC O2-*GFP* | |
| --- | --- | --- | --- |
| *CSF1R*^high^ PBS | *CSF1R*^high^ O2^neg^ | *CSF1R*^neg/low^ O2^pos^ | *CSF1R*^high^ O2^pos^ |
| B1 B3 | B13 B15 | B13 B15 | B13 B15 |
| B2 B10 | B14 B18 | B14 B18 | B14 B18 |
| B4 B9 | B16 B17 | B16 B17 | B16 B17 |
| B11 B12 | B19 B20 | B19 B20 | B19 B20 |
| n = 4 | n = 4 | n = 4 | n = 4 |

**Supplementary Table 3**

Genes in clusters identified through gene-gene MCL cluster analysis (see Figure 4B) and the GO analysis of the clusters are provided in the attached file.

**Supplementary Table 4**

Differentially expressed genes with corresponding expression values and statistical values for analysis of sorted cell populations post APEC O1-*GFP* infection (Sheet 1), APEC O2-*GFP* infection (Sheet 2), and between O1 and O2 infected APEC^pos^ *CSF1R*^high^ cells (Sheet 3) and between O1 and O2 infected APEC^pos^ *CSF1R*^neg/low^ cells (Sheet 4) are provided in the attached file.
